# The draft genome of *Kipferlia bialata* reveals reductive genome evolution in fornicate parasites

**DOI:** 10.1371/journal.pone.0194487

**Published:** 2018-03-28

**Authors:** Goro Tanifuji, Shun Takabayashi, Keitaro Kume, Mizue Takagi, Takuro Nakayama, Ryoma Kamikawa, Yuji Inagaki, Tetsuo Hashimoto

**Affiliations:** 1 Graduate School of Life and Environmental Sciences, University of Tsukuba, Tsukuba, Ibaraki, Japan; 2 Department of Zoology, National Museum of Nature and Science, Tsukuba, Ibaraki, Japan; 3 Center for Computational Sciences, University of Tsukuba, Tsukuba, Ibaraki, Japan; 4 Graduate School of Life Sciences, Tohoku University, Sendai, Miyagi, Japan; 5 Graduate School of Human and Environmental Studies, Kyoto University, Kyoto, Kyoto, Japan; 6 Graduate School of Global Environmental Studies, Kyoto University, Kyoto, Kyoto, Japan; Tierarztliche Hochschule Hannover, GERMANY

## Abstract

The fornicata (fornicates) is a eukaryotic group known to consist of free-living and parasitic organisms. Genome datasets of two model fornicate parasites *Giardia intestinalis* and *Spironucleus salmonicida* are well annotated, so far. The nuclear genomes of *G*. *intestinalis* assemblages and *S*. *salmonicida* are small in terms of the genome size and simple in genome structure. However, an ancestral genomic structure and gene contents, from which genomes of the fornicate parasites have evolved, remains to be clarified. In order to understand genome evolution in fornicates, here, we present the draft genome sequence of a free-living fornicate, *Kipferlia bialata*, the divergence of which is earlier than those of the fornicate parasites, and compare it to the genomes of *G*. *intestinalis* and *S*. *salmonicida*. Our data show that the number of protein genes and introns in *K*. *bialata* genome are the most abundant in the genomes of three fornicates, reflecting an ancestral state of fornicate genome evolution. Evasion mechanisms of host immunity found in *G*. *intestinalis* and *S*. *salmonicida* are absent in the *K*. *bialata* genome, suggesting that the two parasites acquired the complex membrane surface proteins on the line leading to the common ancestor of *G*. *intestinalis* and *S*. *salmonicida* after the divergence from *K*. *bialata*. Furthermore, the mitochondrion related organelles (MROs) of *K*. *bialata* possess more complex suites of metabolic pathways than those in *Giardia* and in *Spironucleus*. In sum, our results unveil the process of reductive evolution which shaped the current genomes in two model fornicate parasites *G*. *intestinalis* and *S*. *salmonicida*.

## Introduction

Reductive genome evolution is most often seen in parasites and endosymbionts that are highly adapted to their niches. Mitochondria, which evolved from an alpha-proteobacterial endosymbiont, is the most representative example of reductive evolution [1]. While genome sizes in the current alpha-proteobacteria are larger than 1Mb in size, the mitochondrial genomes are only several dozens of Kb, indicating drastic genome reduction after endosymbiosis of an ancestral alpha-proteobacterium [1]. Although the last common ancestor of all known extant eukaryotes once experienced harboring mitochondria [2], the sizes and gene repertoires of mitochondrial genomes vary among lineages or even closely related species, suggesting that reductive evolution still occurs in extant eukaryotes [1]. Genome reduction with the loss of functions in parasitic and endosymbiotic lifestyles is supposed to coincide with an increase of dependency on the host.

The Fornicata (fornicates) is a subgroup in a large eukaryotic group Metamonada, which contains two other subgroups called Preaxostyla including *Trimastix* and Parabasalia including *Trichomonas* [3]. All members of Metamonada are known to be adapted to anaerobic or micro-aerobic environments. The model fornicate parasites, *Giardia intestinalis* (the causative agent of beaver fever) and *Spironucleus salmonicida* (the causative agent of pus-filled abscesses in muscles and internal organs in salmonid fish) are closely related to free-living organisms called *Carpediemonas*-like organisms (CLOs) [3–7]. In phylogenetic analyses, free-living CLOs were revealed to be paraphyletic and separately branched at the base of the tree of fornicates, while *G*. *intestinalis* and *S*. *salmonicida* are derived lineages [3].

In fornicates, the genomes of two model parasites, *G*. *intestinalis* and *S*. *salmonicida* are well annotated [8, 9]. In addition, the provisional genome of *Carpediomonas frisia* with the bacterial community was sequenced [10]. Genomes of *G*. *intestinalis* and *S*. *salmonicida* are 11.7 and 12.9 Mbp in size, respectively [8, 9]. The number of protein genes are 5,901 and 8,067, mean lengths of intergenic regions are 481 and 421, and the number of spliceosomal introns in *cis* are 8 and 3 in *G*. *intestinalis* and *S*. *salmonicida*, respectively [8, 9, 11], indicative of small and simple genome structure. On the other hand, despite the small genome sizes and simple structures, abundant gene copies of the cell surface proteins involved in evading host immunity are found in the genomes of the two fornicate parasites. Hundreds of variant-specific surface proteins (VSPs) in *G*. *intestinalis* and cysteine-rich proteins in *S*. *salmonicida* were identified [8, 9]. Only a single variant of those antigenic protein genes is expressed at a time and the expression is switched to another variant in short order [8, 12, 13]. Similar antigenic variant systems are known (or more well-studied) in other parasitic organisms (e.g., *Trypanosoma* and malaria parasites, *Plasmodium*), suggesting a common strategy to avoid the host immune system in independent lineages [14, 15]. Thus, the fornicate genomes especially for parasitic species possess aspects of both genome simplicity and complexity. However, genome evolution in fornicates leading to those parasites remained to be elucidated.

In addition to nuclear genome, reductive evolution in functions of mitochondria is known in various anaerobic eukaryotes [16–18]. And all members of Metamonada including fornicates possess modified versions of mitochondria (so-called mitochondrion-related organelles or MROs) [3]. Although the most representative function of the “canonical” mitochondria is energy production through oxidative phosphorylation, some anaerobic eukaryotes replaced the oxidative phosphorylation pathway by substrate level phosphorylation for the major way of ATP production, presumably through adaptive evolution to an anaerobic lifestyle [16–18]. Besides ATP production, various mitochondrial functions in canonical mitochondria, such as amino acid and iron-sulfur cluster biosyntheses, are known as significant ones for the survival of cells. However, MROs exhibit pronounced reduction of functions compared with canonical mitochondria, and the functions that remain vary among the organisms [3, 16, 17].

Here, we sequenced the genome of a free-living fornicate, *Kipferlia bialata* [19], using a newly established monoxenic culture comprised of *K*. *bialata* and a single bacterial species in order to generate “clean” genome and transcriptome data. We carried out the genomic characterization of *K*. *bialata* and provide an overview of the genome in fornicates. By comparative analysis to the genomes of parasitic close relatives, we modeled reductive genome evolution to parasitic species from a free-living ancestor.

## Material and methods

### Cell cultivation

An original cell culture containing *Kipferlia bialata* NY0173 and feed bacteria was maintained in a 50 ml of liquid culture medium comprised of 90% marine water, 10% mTYGM-9 medium and seven rice grains at 17 °C as previously described [7,19]. Cells were inoculated every 4–7 days to a fresh culture medium.

A monoclonal *Pseudoalteromonas* sp. culture was obtained as described below. The original liquid culture containing *K*. *bialata* and bacteria was streaked on 0.55% marine agar plates (Difco^™^), and the agar plates were incubated for 1–2 days. Single colonies of a bacterium on the plates were picked, and inoculated to 8 ml of liquid culture containing 3.7% marine broth (Difco^™^). Genomic DNAs of mono-bacterial cultures were purified by a modified cetyltrimetyl ammonium bromide (mCTAB) method [20]. 16S rRNA genes of the individual culture strains were amplified by PCR using one of the two sets of universal 16S primers [Forward (5’- AGA GTT TGA TCC TGG CTC AG -3’) + Reverse (5’- TAA GGA GGT GAT CCA GCC -3’), or Forward (5’- CGG CTA ACT MYG TGC CAG CAG CAG CC -3’) + Reverse (5’- GAC TTA ACC CAA CAY YTC AC -3’)]. Amplified PCR products were cloned into pGEM-T easy vector (Promega), and 16S rRNA sequences of each clone were determined by Sanger method using BigDye Terminator v3.1 (ABI). The newly established mono-bacterial culture of *Pseudoalteromonas* sp. was used later as experimental feed for *K*. *bialata*.

### Monoxenic culture establishment

**Fig 1:**
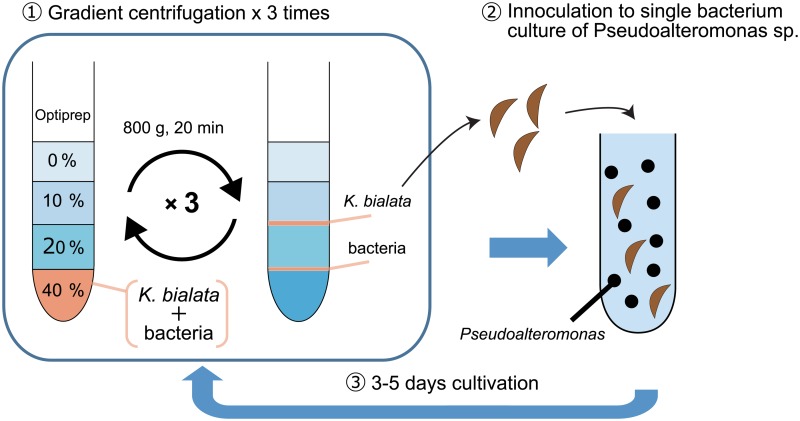
Method summary of the monoxenic culture establishment. The cells with 40% Optiprep was infused at the bottom of tube. And 20, 10 and 0% of Optiprep solutions were stratified on the cell solution. After centrifugation at 800x g for 20 min, *Kipferlia bialata* enriched fraction between 10–20% solutions were collected. Above procedures were repeated three times. *K*. *bialata* enriched fraction were inoculated to the liquid culture contanining single bacterium species, *Pseudoalteromonas* sp. Cells were cultivated 3–5 days. The triple centrifugations followed by cultivation were repeated for 11 times.

The monoxenic culture of *K*. *bialata* containing a single prey bacterial species (*Pseudoalteromonas* sp.) was established as follows. Cells were centrifuged at 800x g for 15 min at 20°C, and the pelleted cells were resuspended in a final concentration of 40% Optiprep (Axis-shield) with 1.25x PBS buffer. The gradient was composed of 4.0 ml each of 0, 10% and 6.0 ml of 20% Optiprep in 2.5x PBS buffer. Cell suspensions were infused at the bottom of the centrifugation tube. Gradients were centrifuged at 800x g for 20 min at 20°C. *K*. *bialata*-enriched fractions between 10–20% Optiprep were collected using a glass pipette, and were suspended again in 75% Optiprep in 2.5x PBS buffer. These density gradient centrifugations were carried out three times. Then, a *K*. *bialata*-enriched fraction from the third round of centrifugation was put into *Pseudoalteromonas* sp. liquid culture, and was cultivated 4–7 days. The triple centrifugations followed by cultivation were repeated for 11 times. The method above is summarized in Fig 1.

### DNA sequencing, assembling and gene annotation

Genomic DNA was purified from the *K*. *bialata*-enriched fraction of the gradient centrifugation by the standard SDS + phenol-chloroform method. Total RNA was also extracted using Trizol (Invitrogen) according to the manufacturer’s instruction. For total RNA extraction, DNase I treatment was done. Purified DNA and RNA were sent to Hokkaido System Science (Hokkaido, Japan) and Eurofin Genomics (Tokyo, Japan), respectively, for TruSeq library construction and Illumina HiSeq 2000 sequencing. After trimming and quality control procedures, genome assembly with 97 million reads (paired-end) was performed using SPAdes with default settings [21], and 54,453 scaffolds were obtained. The scaffolds less than 500 bp in size were discarded. Scaffold lengths with coverage depths were calculated, and two peaks of 10x and 130-150x were observed. The 67 scaffolds with low coverage depth were discarded as bacterial contamination as a result of blastn searches against the NCBI nt database. Additionally, 6 scaffolds of bacterial rRNA operon in the remaining scaffolds were removed. Finally, 11,564 scaffolds (approximately 51 Mbp in total) were assigned as *K*. *bialata* genome sequences. For transcriptome assembling, RNA reads were subjected to Trinity [22], and 20,294 contigs were obtained.

Gene models for the *K*. *bialata* genome were predicted by a combination of genome and transcriptome data using AUGUSTUS as follows. In order to generate parameters, training was performed with genome and transcriptome data using the WebAUGUSTUS server (http://bioinf.unigreifswald.de/webaugustus/about.gsp). AUGUSTUS gene predictions were then carried out with the parameters and ‘hints’ generated during training [23]. Out of the 17,389 predicted protein genes, >97% of genes were supported by the transcriptome data. To assess the genome completeness, BUSCO V3 program with eukaryota_odb9 data set was carried out [24]. Gene annotation, in an automated fashion, was carried out using interproscan [25, 26]. Eukaryotic orthologous group (KOG) assignments were carried out using the WebMGA site (http://weizhong-lab.ucsd.edu/metagenomic-analysis/) with default settings [27]. The genome sequence and raw genome / transcriptome reads of *K*. *bialata* were deposited under DDBJ BioProject PRJDB5457.

### Variant-surface proteins and cysteine-rich protein survey

For the surface membrane protein survey, 253 variant-surface proteins in *Giardia intestinalis* and 415 cysteine-rich proteins in *Spironucleus* were obtained from GiardiaDB (http://giardiadb.org/giardiadb/) and used in blastp or tblastn searches against the *K*. *bialata* predicted protein dataset or the genome including the small scaffolds discarded in the gene prediction procedure (e-value cutoff < 1E-10). Also, the protein genes with annotations of variant-specific surface protein (IPR005127), and growth factor receptor cysteine-rich domain superfamily (SSF57184) were retrieved. The transmembrane domains of those proteins were searched by TMHMM 2.0c [28, 29]. In addition, bacterial surface proteins were searched using *Trichomonas* BspA proteins and pfam motif (PF13306) as query by tblastn (e-value cutoff < 1E-5) or hmm search. All candidate protein hits above were manually curated based on the results of the similarity search against NCBI nr database and domain search.

### Prediction of mitochondrion-related organelle proteins

Candidates for proteins localizing to mitochondrion-related organelles (MROs) in *K*. *bialata* were determined based on phylogenetic relationships to known MRO/mitochondrial proteins. First, to retrieve potential homologous sequences from *K*. *bialata* as well as various organisms, we performed blastp searches against the *K*. *bialata* protein dataset, the NCBI non-redundant (nr) protein database and an in-house comprehensive database comprised of proteins from publicly available genomic and transcriptomic databases, using 31 *Trichomonas vaginalis* and/or *Giardia intestinalis* MRO protein sequences as queries (initial e-value cutoff: 1E-10). Following preliminary phylogenetic analysis by FastTree [30], redundant OTUs were reduced by TreeTrimmer [31] for alignments containing > 140 sequences. If an alignment retained > 140 sequences even after the OTU trimming step, then the procedures were repeated using a blastp search with more stringent e-value threshold. For the final round, multiple alignments for the candidate proteins were generated by MAFFT [32] with the L-INS-i method and poorly aligned sites or gaps were automatically removed from the final datasets by trimAl [33]. Finally, maximum likelihood trees were inferred by IQ-TREE [34] with ultrafast bootstrapping [35] under substitution models selected by the model test tool implemented in IQ-TREE for each dataset.

In cases where homologs were not detected due to very short protein sequences (truly short or truncated) using the above mentioned automated phylogenetic analysis, blast searches against *K*. *bialata* genome and transcriptome data were manually carried out. Top hits were then manually curated based on the protein domain structure and the similarity to known proteins (e.g., homologs of bacteria).

The presence of mitochondria-targeting signals in the proteins in question were evaluated using three different programs—MitoFates, TPpred2 and TargetP and IPSORT [36–40]. The N-termini of the predicted proteins were manually curated using the genome and transcriptome data. If N-termini of the proteins predicted from genome data were truncated, the N-termini of corresponding transcript sequences including splicing variants were subjected to the mitochondrial-targeting signal prediction analysis.

## Results and discussion

### Establishment of the monoxenic culture useful for genomic analyses

**Fig 2:**
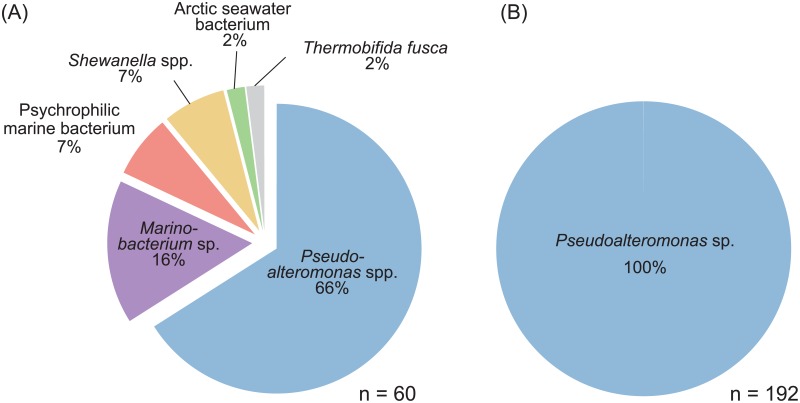
The bacterial composition. (A) the original and (B) the monoxenic culture analyzed in this study. “*n*” indicates the numbers of the 16S rDNA clones sequenced.

For genomic, transcriptomic and/or proteomic research of non-axenic culture strains, especially bacteria-feeding micro eukaryotes, exclusion of possibility for contamination from food bacteria is always a challenging issue if the culture contains multiple bacteria of which species composition is unknown [10, 41, 42]. In order to obtain clean genome and transcriptome data, we established a monoxenic culture comprising *Kipferlia bialata* and a single bacterium. Briefly, the combination of the density gradient centrifugation and the inoculation of *K*. *bialata* cells to *Pseudoalteromonas* sp. culture was carried out 11 times (Fig 1). In order to evaluate the bacterial composition in the culture, genomic DNA was purified and bacterial 16S rRNA sequences were determined by cloning and Sanger sequencing. Although the original culture contained at least six different bacterial species (*n* = 60), only *Pseudoalteromonas* sp. sequences was detected (*n* = 196) after the above mentioned procedures (Fig 2). This newly established culture was used in later analyses.

**Fig 3:**
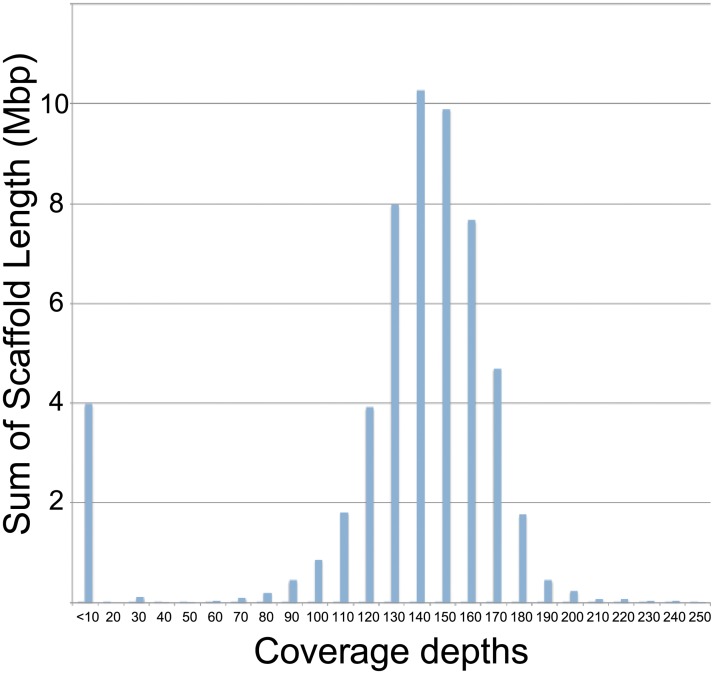
Sequencing depths analysis of the *Kipferlia bialata* genome. Coverage depths distribution of the original scaffolds before bioinformatic filtration. Coverages were calculated based on the SPAdes output. Scaffolds were generated using 200 million paired-end reads (half of available reads).

The genomic DNAs and the mRNAs from the *K*. *bialata*-enriched fraction were subjected to Illumina Hi-Seq platform sequencing. After genome assembly, two peaks of coverage depths were observed in 10x and 130-150x coverage positions (Fig 3). The total length of genome scaffolds with less than 30x coverages (strictly 2-15x, no scaffolds with 16-29x) were 3.98 Mbp which is similar size to the full genome size of those top blastn hit, *Pseudoalteromonas* SM9913 (4.0 Mbp in size). Thus, the scaffolds with low coverage depths were discarded in later analyses as the scaffolds for the food bacterium. Bacterial rRNA sequences in remaining scaffolds were also excluded based on similarity searches. Finally, 51 Mbp of genome scaffolds with approximately 150x coverage were assigned to *K*. *bialata* genome scaffolds. The total genome scaffold size is consistent with the genome size prediction based on kmer frequency analysis (i.e., 49–54 Mbp). Additionally, kmer frequency analysis showed two peaks of kmer abundance, suggesting that *K*. *bialata* contains a diploid nuclear genome.

The procedures we applied here were highly efficient to exclude any bacterial cells other than one species *Pseudoalteromonas* sp., which we chose as a food bacterium for *K*. *bialata*. Since the procedures are simple and only require an ordinary centrifugation machine and a few commercial reagent products, we believe the procedures are useful to establish monoxenic cultures for many other microbial eukaryotes and would contribute to their genome and proteomics analyses.

### Overview of *Kipferlia bialata* genome

An overview of the *K*. *bialata* genome and of the fornicate parasites and *Trichomonas vaginalis* genomes is shown in Table 1. The BUSCO output which is a guide index of genome completeness is shown (S1 Fig). The coverage of core protein hits of *K*. *bialata* was similar level to the well annotated genome of *G*. *intestinalis*. Approximately 35% of protein hits were fragmented, because those fragmented proteins are located at the edges of the genome scaffolds. We therefore used both the genome and the transcriptome data for later functional analyses (see below section). G+C content and the number of protein genes in *K*. *bialata* were 49.4% and 17,389, respectively. In comparison with fornicate parasites, *G*. *intestinalis* and *S*. *salmonicida*, the free-living *K*. *bialata* genome is larger in terms of size, the number of protein genes and the coding capacity (i.e., longer intragenic distance and high gene density) (Table 1). The most significant structural difference found in the *K*. *bialata* genome when compared with the genomes of fornicate parasites is the intron abundance. While a small number of *cis*-introns exist in the *G*. *intestinalis* and *S*. *salmonicida* genomes (8 and 3, respectively) [8, 11], 124,912 canonical introns with GU-AG boundary were found in the *K*. *bialata* genome (Table 1). Since *K*. *bialata* is the only species with abundant introns in the fornicate clade to date, it is possible that the numerous introns were independently obtained on the line leading to *K*. *bialata*. However, secondary loss of introns in the evolution leading to a parasitic lifestyle in *G*. *intestinalis* and *S*. *salmonicida* is the more likely scenario because i) secondary intron loss was reported across the different *Giardia* strains [11]. More broadly, ii) parasitic genomes tend to lose introns in general [43], iii) the intron density of *K*. *bialata* (2.45 introns per kbp) is not especially high compared with genomes from other free-living eukaryotic organisms (e.g., 1.6–7.8 in animals, 0.6–6.1 in archaeplastida, 0.8–4.3 in heterokonts + alveolates) [44] and iv) an eukaryotic ancestor (not limited to fornicates) is inferred to have had an intron-rich genome [44]. These observations suggest that fornicate parasites secondarily lost their introns. It is also known that the genome of *T*. *vaginalis* which belongs to Parabasalia, sister to fornicates, possesses spliceosomal introns as few as *G*. *intestinalis* and *S*. *salmonicida* (Table 1) [45]. If the number of introns in *K*. *bialata* reflects an ancestral state of genomes in Metamonada, drastic losses of spliceosomal introns have happened independently in parasitic Metamonada species, possibly associated with adaptation to parasitic lifestyles. On the other hand, since the genome data of free-living fornicates are currently limited, more genome data are necessary to confidently gain insight into whether the adaptation to parasitic lifestyle is connected to general genome reduction such as reduced genome size and the loss of the introns in fornicates.

**Table 1:** Overview of nuclear genome sequences for Metamonada species.

Species	*Kipferlia bialata*	*Spironucleus salmonicida*	*Giardia intestinalis*	*Trichomonas vaginalis*
**Genome size (Mbp)**	**51.0**	**12.9**	**11.7**	**176.4**
**G+C content (%)**	49.4	33.4	49.0	32.7
**Number of protein genes**	17,389	8,067	5,901	25,949*
**Mean amino acid length (amino acids)**	333.0	373.0	530.0	309.5
**Mean intergenic distance (bp)**	596.5	421.0	481.0	1165.4
**Gene density (gene / kbp)**	0.34	0.63	0.50	0.15
**Number of spliceosomal introns**	124,912	3	8	65
References	In this study	[8]	[8,9,11]	[45]

*Only evidence-supported gene number is shown (see results in [45]).

### Complexity of cellular functions

**Fig 4:**
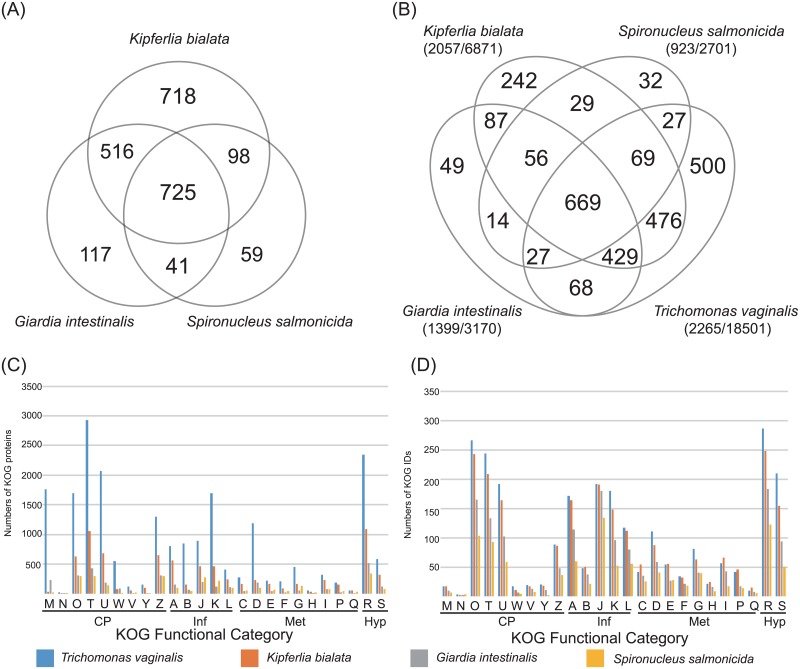
Functional annotation based on the eukaryotic orthologous groups (KOG). (A) Venn diagram showing the number of shared unique KOG identifiers (IDs) under three-ways comparison amongst fornicates including *Kipferlia bialata*, *Giardia intestinalis* and *Spironucleus salmonicida* and (B) the four-ways comparison considering with *Trichomonas vaginalis*. The number under species names indicates the total number of unique KOG IDs (left) and the total number of proteins assigned into KOG (right). (C) Histogram showing the number of proteins assigned into KOGs and (D) the unique number of KOG IDs in each genome. KOG categories are as follows: A, RNA processing and modification; B, chromatin structure and dynamics; C, energy production and conversion; D, cell cycle control, cell division and chromosome partitioning; E, amino acid transport and metabolism; F, nucleotide transport and metabolism; G, carbohydrate transport and metabolism; H, coenzyme transport and metabolism; I, lipid transport and metabolism; J, translation, ribosomal structure and biogenesis; K, transcription; L, replication, recombination and repair; M, cell wall, membrane or envelope biogenesis; N, cell motility; O, post-translational modification, protein turnover, chaperones; P, inorganic ion transport and metabolism; Q, secondary metabolites biosynthesis, transport and catabolism; R, general function prediction only; S, function unknown; T, signal transduction; U, intracellular trafficking, secretion and vesicular transport; V, defence mechanisms; W, extracellular structures; Y, nuclear structure; Z, cytoskeleton. Higher KOG categories are as follows: CP, cellular processing and signaling; Hyp, poorly characterized; Inf, information storage and processing; Met, metabolism.

To assess complexity of cellular functions encoded in fornicate genomes, we focus on the protein function assignments based on the eukaryotic orthologous groups (KOG). The variety of the KOG identifiers (IDs) found in *K*. *bialata* is more abundant compared with those of *G*. *intestinalis* and *S*. *salmonicida* (Fig 4). The number of KOG IDs found in each fornicate genome is 2,057 (6,871 proteins), 1,399 (3,170 proteins) and 923 (2,701 proteins) in *K*. *bialata*, *G*. *intestinalis* and *S*. *salmonicida*, respectively (Fig 4A and 4B). In the three-ways comparison amongst fornicate genomes, 718 KOGs were found to be unique in *K*. *bialata* genome (Fig 4A), suggesting a reduction of general functions in fornicate parasites after the branching from the line leading to *K*. *bialata*. Despite of the reduced number of KOG IDs, the proteins assigned into cell wall, membrane or envelope biogenesis (M) and extracellular structures (W) were found to be exceptionally abundant in fornicate parasites. In the KOG classes, 41 and 237 proteins were assigned to M, and 77 and 90 proteins were assigned to W in *K*. *bialata* and *G*. *intestinalis*, respectively (Fig 4C), suggesting that surface proteins in *G*. *intestinalis* is more complex than those in the free-living relative. While the numbers of unique KOG IDs for *G*. *intestinalis* were 7 (M) and 5 (W), these numbers were fewer than those for *K*. *bialata* [10 (M) and 7(W);Fig 4D]. Thus, although the number of proteins in M and W categories for *G*. *intestinalis* tends to be larger relative to the total number of protein genes presumably due to multiple copy genes, the number of IDs/functions appeared to be restricted. Presumably the dispensable functions for parasitic lifestyle disappeared from parasitic fornicates, although the clear evidence of the loss of functions/pathways relating parasitic lifestyle were undetectable in this study. The future study based on the genome and the experimental data could unveil the relation between loss of general functions and parasitic lifestyle.

While parasitic fornicates retain less number of proteins with general functions compared with free-living *K*. *bialata*, *T*. *vaginalis* (Parabasalia) were found to contain more abundant general proteins than fornicates. (Fig 4B, 4C and 4D). Nevertheless, the direct comparison between *K*. *bialata* and *T*. *vaginalis* genomes, and the discussion for their common genome architecture, are presumably difficult at this moment, because the genome expansion of *T*. *vaginalis* is supposed to be occurred recently after the species split [45]. The genome size of *T*. *vaginalis* is approximately 180 Mbp, larger than the genome of *K*. *bialata*, and this genome size is large in general compared with well-known medically important parasites other than Metamonads (e.g., *Plasmodium* and *Entamoeba*) [46, 47]. Also, *T*. *vaginalis* contains up to approximately 60,000 protein genes including transposable elements, repeats and the 25,949 evidence-supported genes with either similar to the known proteins or with expressed sequence tags [45, 48]. Thus, the genome evolution of *T*. *vaginalis* might be an exceptional case in the adapting process to parasitic lifestyle.

In *G*. *intestinalis*, variant surface proteins (VSPs) are supposed to constitute one of the most vital mechanisms of parasites for the evasion of the host immune system. Abundant variants were found to be encoded despite its highly reduced genome [12, 13]. *S*. *salmonicida* also possesses abundant cysteine-rich proteins (CRPs) showing affinity to the VSPs [8]. Although we identified the surface membrane proteins, 253 VSPs of *G*. *intestinalis* and 415 CRPs of *S*. *salmonicida* in GiardiaDB (http://giardiadb.org/giardiadb/), neither hits showing significant similarity to VSPs nor to CRPs were detected in the *K*. *bialata* genome (e-value cutoff < 1E-10). We also searched against the transcriptome data, but no significant homologs were found.

To survey the surface proteins more broadly, we retrieved the proteins with cysteine rich domain (INTSSF57184) from interproscan annotation in *K*. *bialata*, *G*. *intestinalis* and *S*. *salmonicida*. While 248 proteins (including 199 VSPs: IPR005127), 232 of which are with predictable transmembrane domain, and 327 proteins, 279 of which with transmembrane domain, were detected in *G*. *intestinalis* and *S*. *salmonicida*, respectively, only 19 proteins with cysteine rich domain were found from *K*. *bialata*. In addition, although 11 of 19 proteins with cysteine rich domain were predicted to contain the transmembrane domain, neither CxxC nor CxC motifs, characteristic for VSPs and CRPs, were detected.

In addition to fornicate parasites, the cell surface of *T*. *vaginalis* is also covered by highly variable proteins, although their functions still remain unclear [49]. The largest surface protein family, *Bacteroides* surface protein A (BspA), was found to have been acquired via lateral gene transfer in *T*. *vaginalis* [50,51]. In the tblastn and hmm search, only three BspA homologs were found from the *K*. *bialata* genome and transcriptome. However, since they showed no significant similarity to BspA of *T*. *vaginalis*, *K*. *bialata* (or its ancestor) was supposed to acquire the BspA independently by lateral gene transfer from bacteria after split from the line leading to Parabasalia.

In any case, *K*. *bialata* possess fewer surface protein homolog repertory compared with parasites in question. These observations are in agreement with the view that increase of surface protein complexity is involved in the evolution of parasitism. The major reason for the difference of the number of KOG proteins M and W between fornicate parasites and *K*. *bialata* was suggested to be the absence of cell surface proteins for evasion of host immunity such as VPSs and CRPs from *K*. *bialata*. Fornicate parasites including *G*. *intestinalis* and *S*. *salmonicida* might have newly acquired the abundant surface proteins throughout the evolution to parasitism after divergence from a common ancestor with *K*. *bialata*.

### Reconstructed metabolic pathways localized in mitochondrion-related organelles of *Kipferlia bialata*

Anaerobic eukaryotes often lack canonical mitochondria capable of performing aerobic respiration but possess mitochondrion-related organelles (MROs) that have evolved from aerobic mitochondria in reductive manners during adaptation to anaerobic environment [16–18]. Recently, the metabolic pathways of MROs in various CLOs including *K*. *bialata* was reported [3]. However, their prediction was solely based on transcriptome analyses [3]. Due to difficulty in determining full length of coding-sequences by transcriptomic analyses, sequences of some proteins such as those for pyruvate metabolisms, lacked N-terminal sequences and localization of such pathways remained to be more accurately elucidated. Thus, the necessity of supporting survey based on the genomic information was pointed out [3]. On the basis of the consensus between the genome and transcriptome data of *K*. *bialata*, we successfully reconstructed more accurate MRO functions. The prediction of MRO functions in *K*. *bialata* was carried out based on a representative variety of function known MRO proteins in *T*. *vaginalis* and *G*. *intestinalis*. Twenty-eight and six proteins, targeted to *T*. *vaginalis* or *G*. *intestinalis* MROs, respectively, were taken as query sequences in similarity searches (Table 2). In addition, the five proteins related to substrate-level phosphorylation and the four proteins involved in the antioxidant defenses of *G*. *intestinalis*, which are functional homologs of *T*. *vaginalis* MRO proteins (but localized in the cytosol), were searched for as well (Table 2). MRO protein homologs were judged by the results of similarity search and phylogenetic analyses (see Material and methods and S2 Fig). All candidates including splicing variants were retrieved from the genome and transcriptome data, and presence or absence of N-terminal mitochondrial targeting sequences were surveyed. The homologs of all proteins listed in Table 2 including four proteins for iron-sulfur cluster biosynthesis and three ISC transporters, seven proteins of the glycine cleavage system, 11 proteins of the anaerobic ATP synthesis pathway and six proteins involved in anti-oxidant defenses, were identified in the *K*. *bialata* genome. In addition, ASCT B and GCSP proteins, which are absent from *T*. *vaginalis* and *G*. *intestinalis* MROs, were detected in the *K*. *bialata* genome. It was suggested that free-living species in Metamonada including CLOs possess split GCSP proteins called GCSP1 and GCSP2 corresponding to domain1 and domain2, respectively [3, 52]. Consistent with the finding, the GCSP protein of *K*. *bialata* was confirmed to be encoded as two split genes, and transcribed separately, on the basis of our protein survey for genome and transcriptome using manual curation combined with phylogenic analyses (Table 2 and S2 Fig).

**Table 2:** Protein list surveyed in this study.

Protein	*Trichomonas*	*Giardia*
**Iron-sulfur cluster biosynthesis and transport**		
IscU^¶^	XP_001580655	EFO61914
IscS^¶^*	XP_001320829	EFO64129
IscA*	XP_001316269	EET01458
Frataxin	XP_001583965	-
Hsp70^¶^*	XP_001582674	XP_001710092
HscB	XP_001308237	EFO65039
GrpE^¶^*	XP_001329309	XP_001706621
**Glycine cleavage system**		
SHMT^¶^*	XP_001322593	-
GCST*	-	-
GCSP1^¶^*	-	-
GCSP2^¶^*	-	-
GCSH^¶^*	XP_001299513	-
GCSL^¶^*	XP_001330332	-
NuoE^¶^	XP_001312168	-
NuoF*	XP_001321469	-
**Anaerobic ATP synthesis**		
ME^¶^*	AAA92714	XP_001707581
PFO^¶^*	AAA85495	EET01691
ferredoxin^¶^	XP_001324407	ABV81956
Hydrogenase^¶^*	XP_001305709	EFO64299
HydE^¶^*	XP_001326754	-
HydF^¶^*	XP_001309182	-
HydG*	XP_001313153	-
ASCT B^¶^*	-	-
ASCT C^¶^	XP_001330176	-
ACS	-	EET01173
SCSa^¶^*	XP_001328129	-
SCSb	XP_001303981	-
**Antioxidant defenses**		
Trx*	EAY13577	EES99386
TrxR	XP_001316923	EET01934
TrxP	EAY01083	ESU45818
SOD^¶^*	EAY08252	-
HCP*	AAL66298	AAL66297
Rbr*	XP_001320125	-

The list of MRO-related proteins. All proteins shown were identified from *Kipferlia* genome. The proteins related to mitochondrial functions follow [3] and [16].

^¶^ shows that mitchondrial-targeting signals were detected by at least one prediction program.

* indicates multi copy genes in *Kipferlia* genome.

Of the *K*. *bialata* proteins corresponding to *T*. *vaginalis* MRO protein homologs, 6 of 7 iron-sulfur cluster biosynthesis + transporter proteins, 5 of 7 protein homologs of the glycine cleavage system and 9 of 10 proteins involved in anaerobic ATP synthesis were predicted to contain mitochondrial-targeting signals by at least one prediction program (Table 2). The detected mitochondrion-targeting signals for the majority of the proteins in each system suggest that iron-sulfur cluster biosynthesis, the glycine cleavage system and the anaerobic ATP synthesis pathway exist in the *K*. *bialata* MRO. On the other hand, only 2 of 6 proteins relating to the anti-oxidant defenses were predicted to have mitochondrion targeting. Therefore, it is unclear whether the anti-oxidant defense functions are partially or fully localized in the MRO. It is worth noting that even if “typical” mitochondrial targeting signals are absent, it does not always mean the proteins are localized to the cytosol in MRO-bearing organisms. MRO proteins without “typical” targeting signals have been reported in *T*. *vaginalis*, *G*. *intestinalis* and microsporidians [53–55]. Indeed, mitochondrial cpn60 protein in *K*. *bialata* lacked predictable mitochondrial targeting signal despite 5’UTR were recognized. If the anti-oxidant defenses were localized in the *K*. *bialata* MRO, the role of the MRO in *K*. *bialata* is very similar to that in *T*. *vaginalis*. In addition to the predicted MRO proteins, ATP transporters with transmembrane domains potentially locate on MRO membrane were listed (S1 Table).

**Fig 5:**
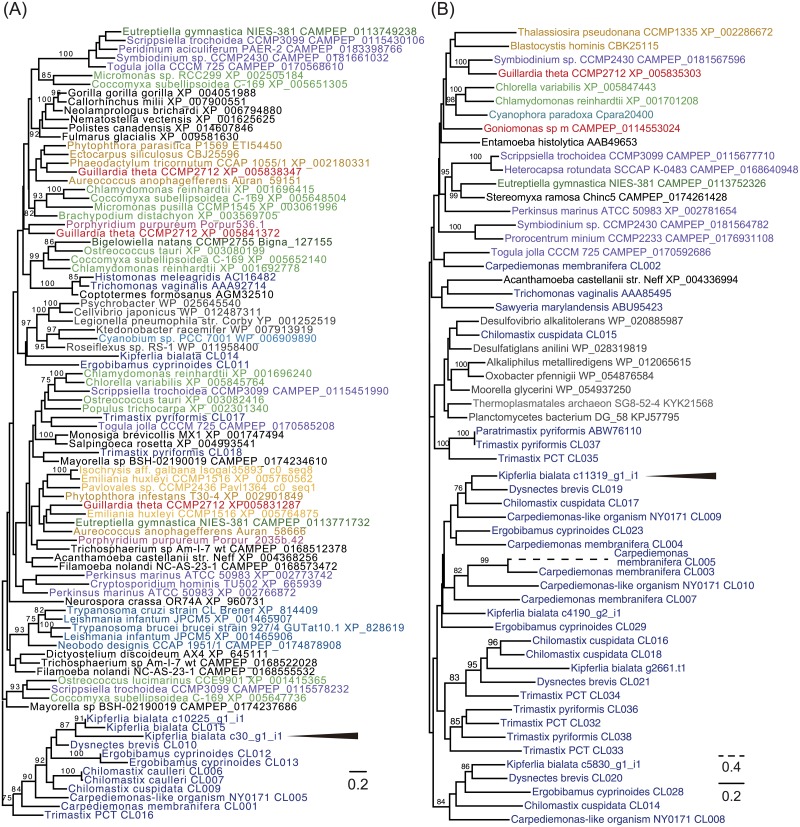
Phylogenetic analyses. Maximum-likelihood tree inferred from (A) malic enzyme and (B) pyruvate:ferredoxin oxidoreductase. Trees were inferred by using IQ-tree with LG4X model. Numbers above branches are bootstrap support values. Bootstrap support values higher than 75% are shown. Arrowheads indicate the mitochondrial-targeting protein predicted by at least one prediction program.

Basically, our survey on the *K*. *bialata* genome showed consistent result with previous work based on previous transcriptome data with a few exceptions [3]. Although HydE was not detected in the previous study, HydE was retrieved from our genome data. In addition, SHMT was confirmed to have a predictable mitochondrial-targeting signal. Thus, major MRO functions in *K*. *bialata* were revised by analyzing the genome data which was absent [3]. Another finding in this study is of pyruvate metabolism concomitant with ATP synthesis, of which localization was not precisely predicted in the previous study. *K*. *bialata* possesses ACS, a homolog of cytosolic ATP synthase in *G*. *intestinalis*, in addition to mitochondrial ASCT and SCS (*Trichomonas* type; see also [3]). This suggests that *K*. *bialata* synthesizes ATP through substrate-level phosphorylation in two separate cellular compartments, namely the cytosol with ACS (*Giardia* type) and the MRO with ASCT + SCS (*Trichomonas* type). Both pathways require acetyl-CoA as a substrate for ATP synthesis. The existence of multiple copies of the malic enzyme (ME), pyruvate ferredoxin oxidoreductase (PFO) and a component of hydrogenase, which play a role in serving acetyl-CoA synthesized from malate, supports the presence of anaerobic ATP synthesis pathways in *K*. *bialata* (Table 2). The protein varieties of ME and PFO with/without mitochondrial targeting signals further supports the ATP synthesis in multiple cellular compartments, because these homologs presumably correspond to the cytosolic and MRO-targeted types (Fig 5).

**Fig 6:**
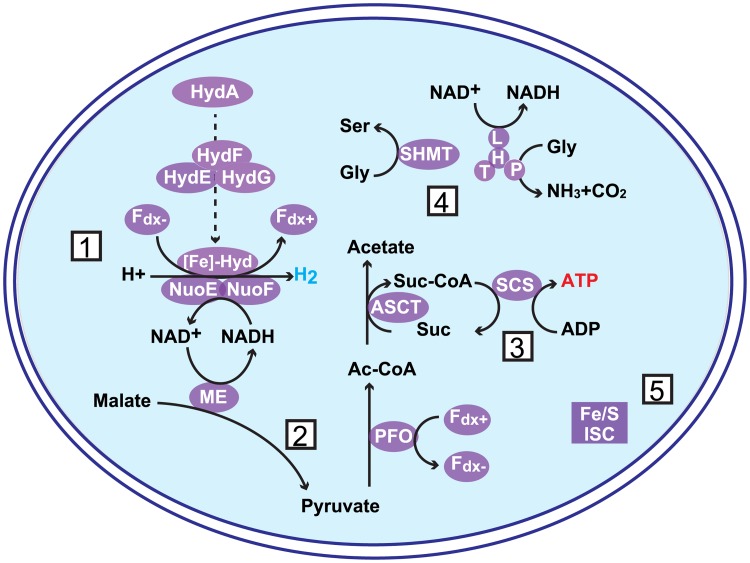
Reconstructed metabolic pathway of mitochondrion-related organelles in *Kipferlia bialata*. 1. H_2_-synthesis, 2. Pyruvate metabolism, 3. Substrate-level phosphorylation, 4. Amino acid metabolism, and 5. Fe-S cluster assembly. NuoE and F: 24 and 51 kDa of mitochondria NADH:ubiquinone oxidoreductase, respectively, Fe-Hyd: Fe-hydrogenase, HydE/F/G: Fe-hydrogenase maturases, Fdx: ferredoxin, ME: malic enzyme, SHMT: serine hydroxymethyltransferase, H/L/P/T: glycine cleavage system proteins H/L/P/T, Fe/S ISC: iron-sulfur cluster assembly ISC system, SCS: succinyl-CoA synthase, ASCT: acetate:succinyl-CoA transferase, PFO: pyruvate:ferredoxin oxidoreductase. Suc: Succinate, Ac-CoA: Acetyl-Coenzyme A, suc-CoA: succinyl-Coenzyme A.

It is worth mentioning that the MRO functional survey in this study is limited to the proteins found in *T*. *vaginalis* and *G*. *intestinalis*. Nevertheless, we could identify more complete metabolic pathways probably localized in MROs of *K*. *bialata*, than predicted in the previous study (Fig 6) [3]. Although the *K*. *bialata* MROs are assumed to possess additional functions (e.g., malate import for pyruvate metabolism), we were not able to identify them by the similarity-based search. To reveal the whole *K*. *bialata* MRO function, we need to deepen our knowledge on the MRO functions in diverse anaerobic eukaryotes by future studies presumably based on proteomic data.

## Conclusion

In the present study, we revealed that reduced genomes of the current fornicate parasites have evolved from the ancestral genome which was larger in size, richer in gene repertory and more abundant in introns. In contrast, the complexity of surface proteins was increasing and specializing to accommodate a parasitic lifestyle in the evolution leading to *Giardia*/*Spironucleus*. Thus, genome reduction and an increase of the surface protein complexity appear to have occurred simultaneously in fornicate parasites. The possible explanation for this inconsistency between gain and loss is that an increase in the surface proteins contributed to an increase in success of evading the host’s immune system, while a resulting reliance on a parasitic lifestyle enabled the parasites to dispense with genes for proteins/functions/byproducts provided by the host. A streamlined genome was the trade-off for increased specialization. This loss of function with a gain of complexity in some areas is probably not limited to fornicate parasites but also found in other parasitic lineages such as trypanosomatids and apicomplexans [14, 15].

## Supporting information

S1 Fig BUSCO assessment results. (PDF)

S2 Fig Phylogenetic trees inferred from mitochondrion-related organelle proteins. (PDF)

S1 Table Predictable ATP transporters in *Kipferlia bialata*. The left column indicates the protein IDs of *K*. *bialata* ATP transporter candidates. The second left column shows the number of transmembrane domains predicted by TMHMM 2.0c. Remaining columns display results of the tblanstn search. Each of the query proteins from human or mouse used for the search is shown in the top cell. log 10 of the e-value for the hit between the query and each of the *K*. *bialata* proteins is shown in the corresponding cell. Only the hits with e-value < 1e-15 are shown.
(PDF)
