# Supplementary figures and images for: The draft genome of Kipferlia bialata reveals reductive genome evolution in fornicate parasites
Source: PLoS One. 2018 Mar 28;13(3):e0194487. doi: 10.1371/journal.pone.0194487 (PMC5874029; doi:10.1371/journal.pone.0194487)

# BUSCO Assessment Results

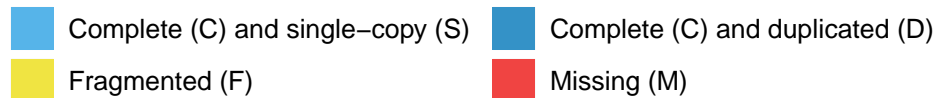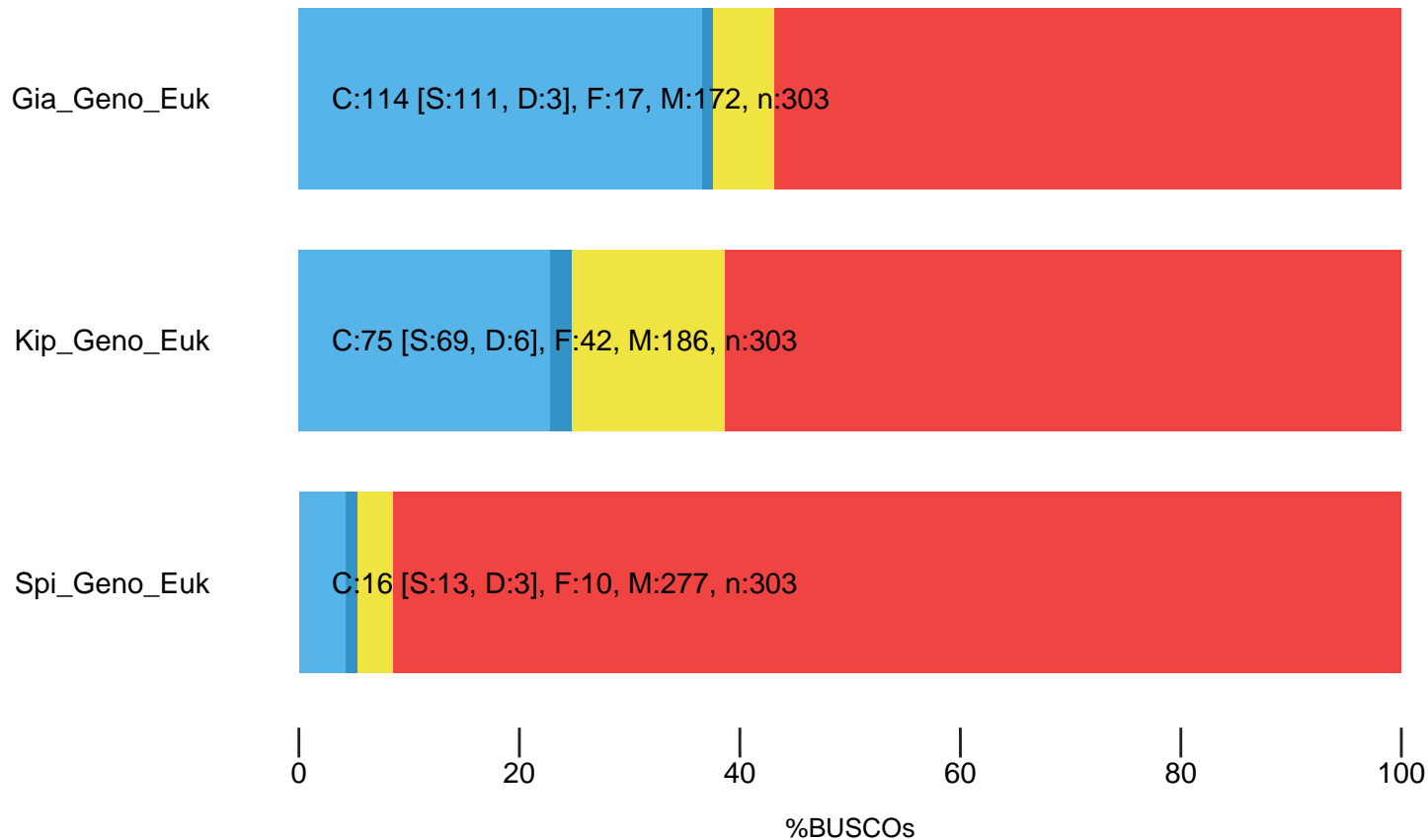

Supplement: S1 Fig — (PDF) [file pone.0194487.s001.pdf]

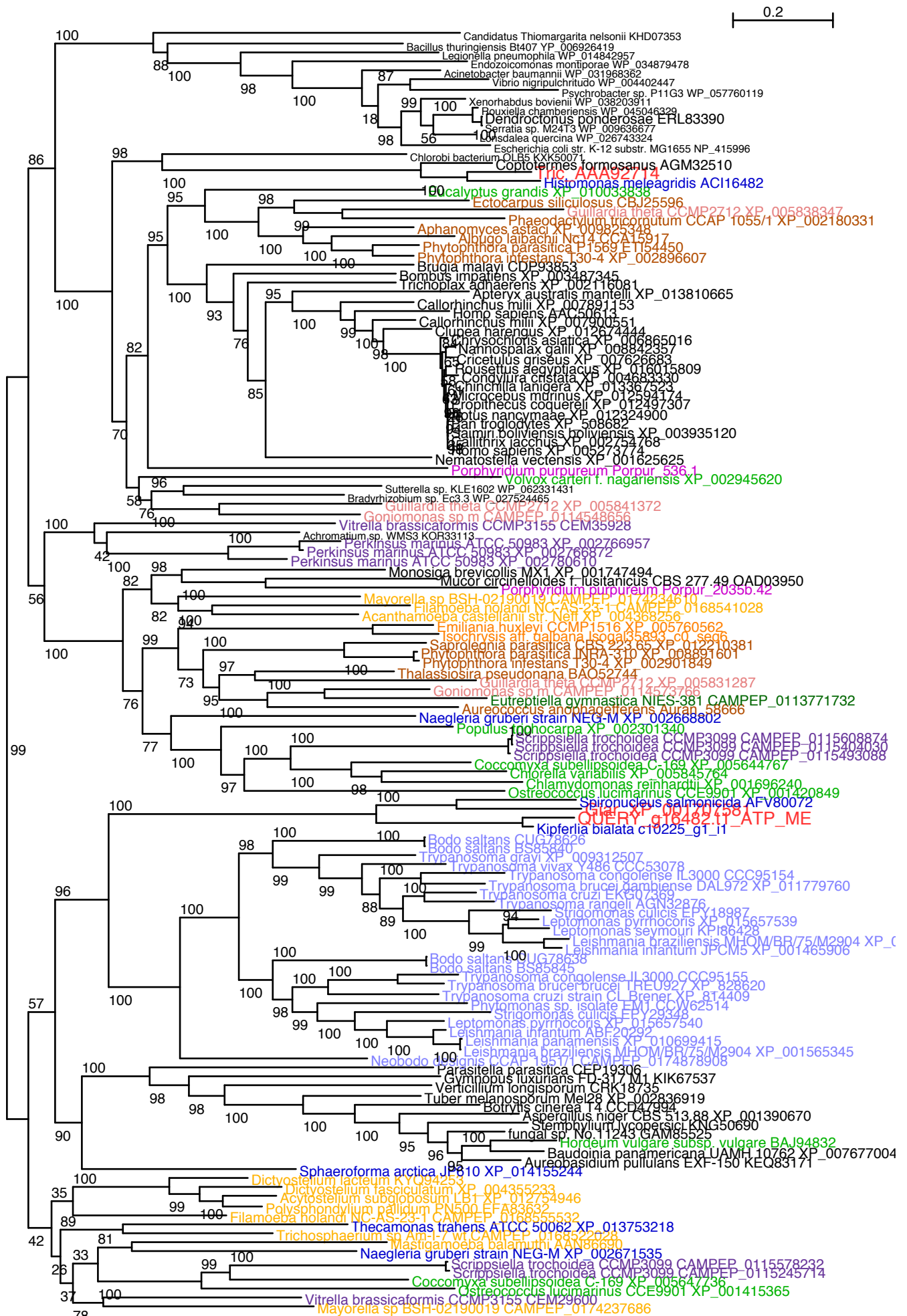

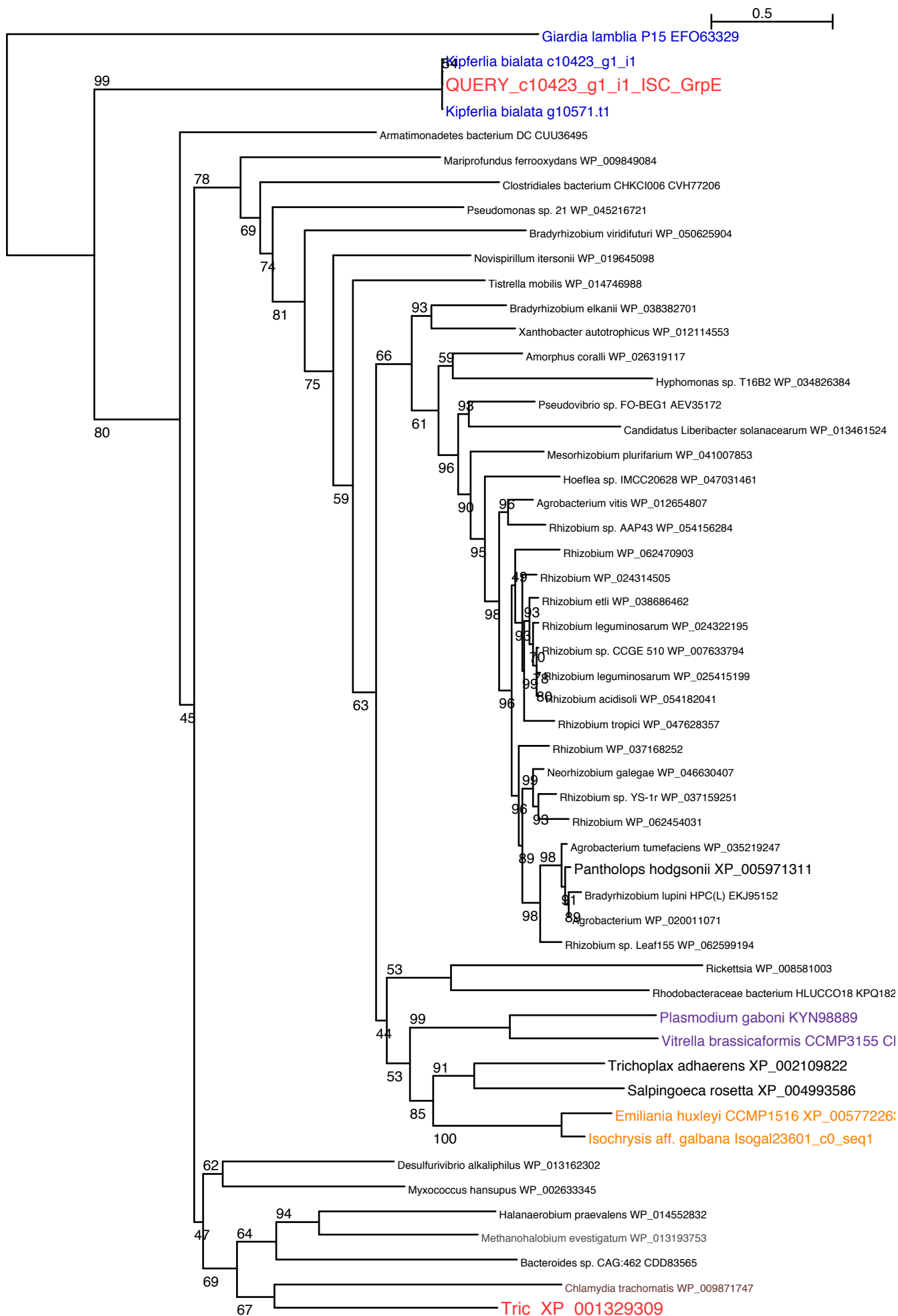

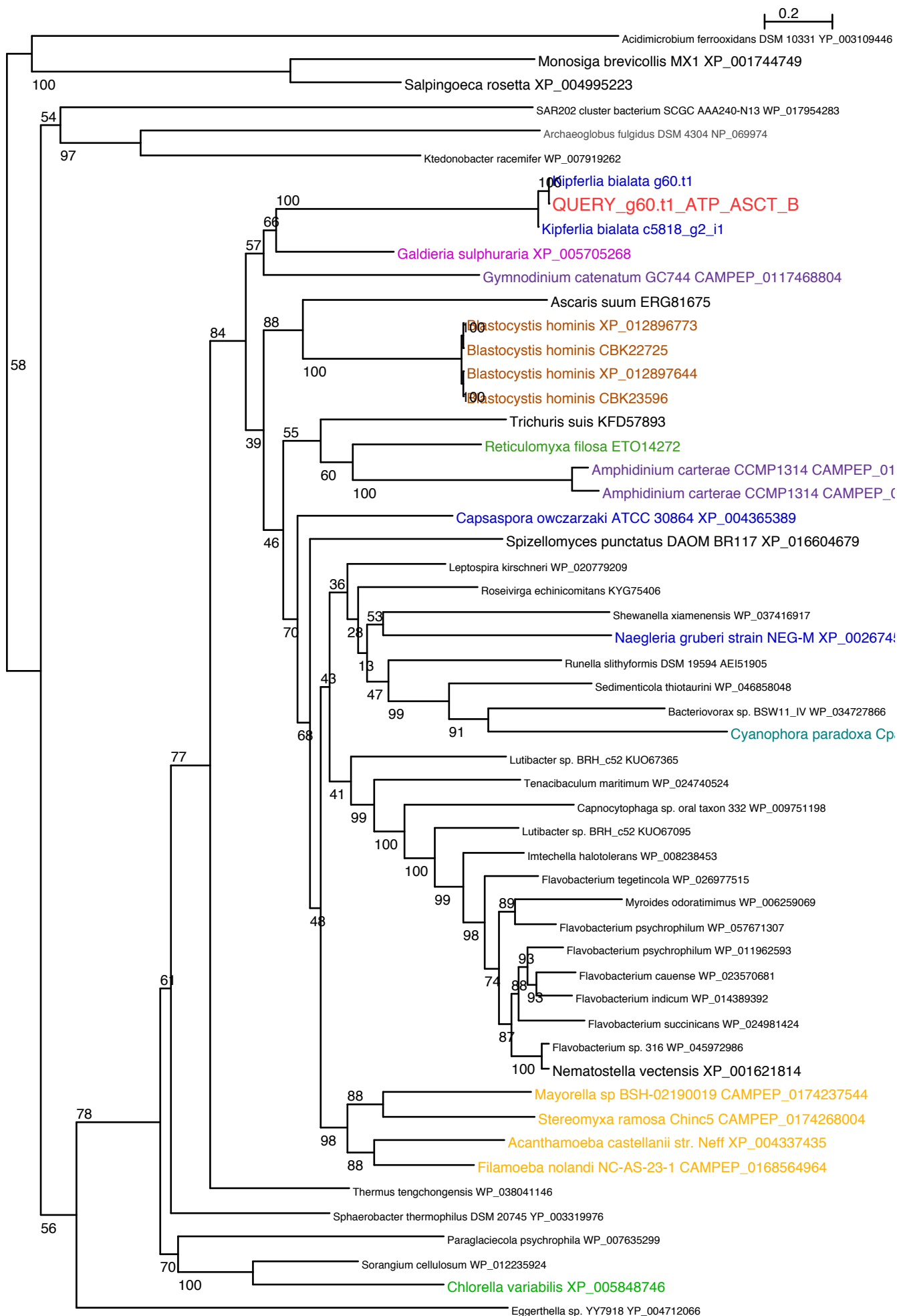

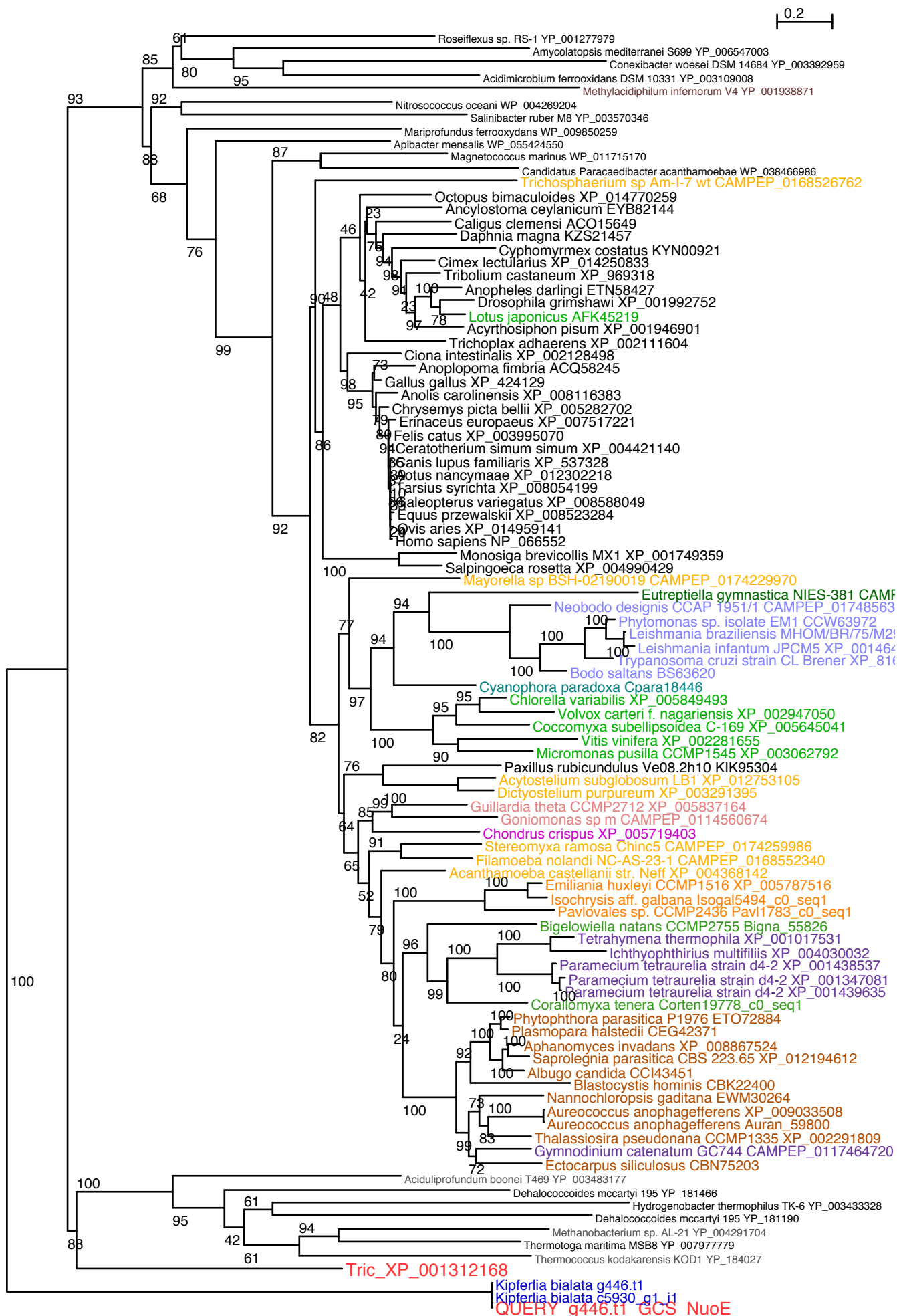

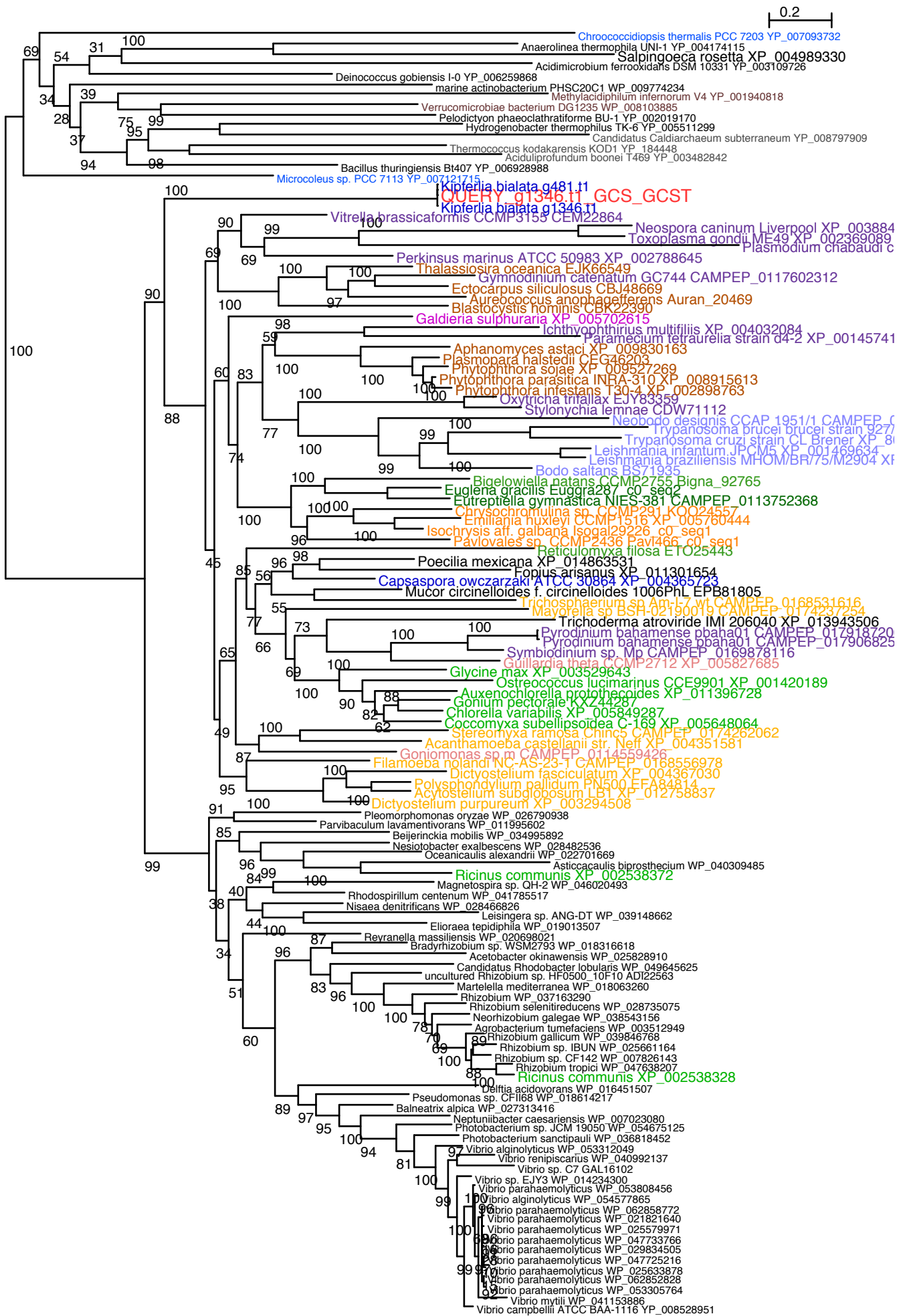

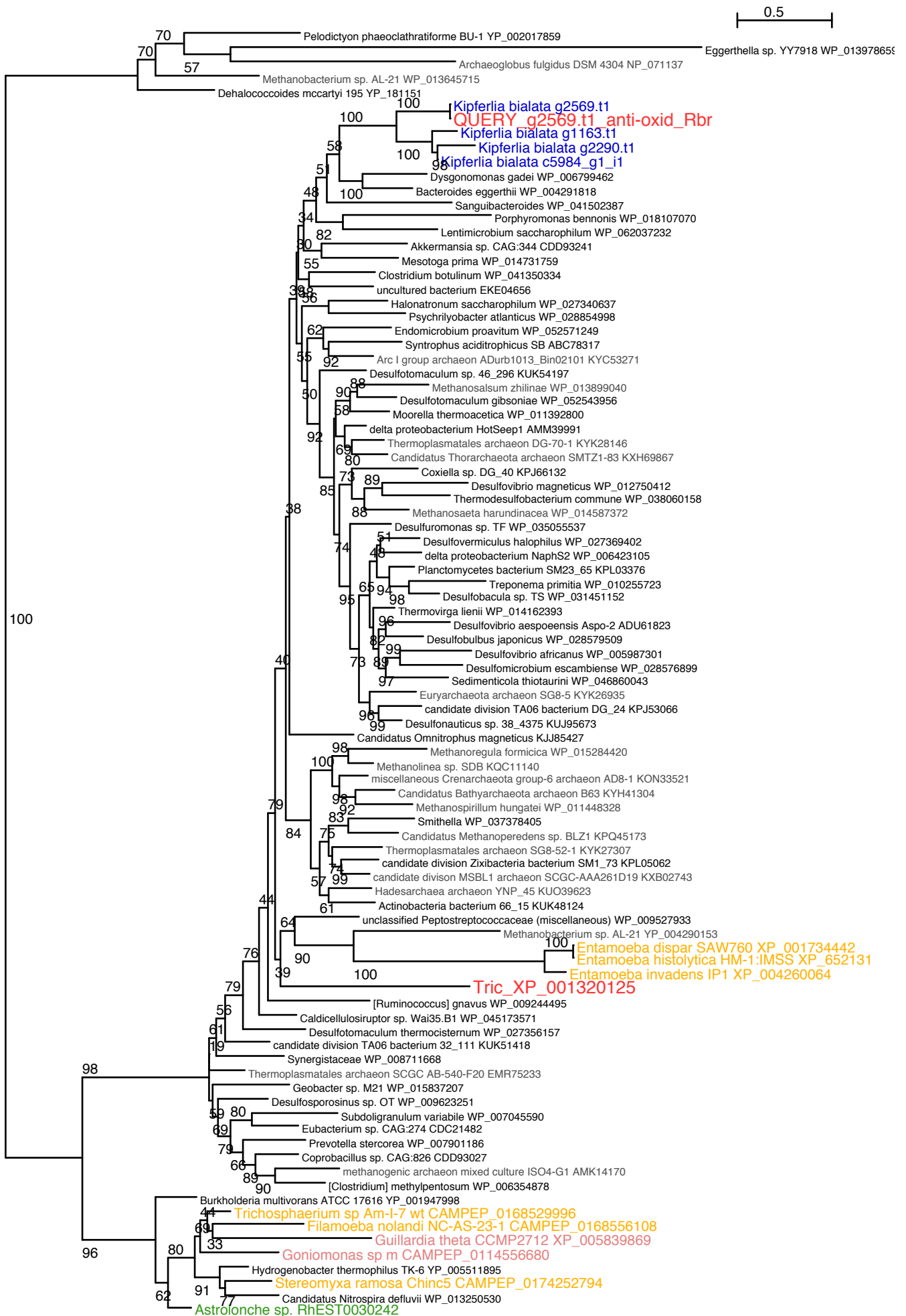

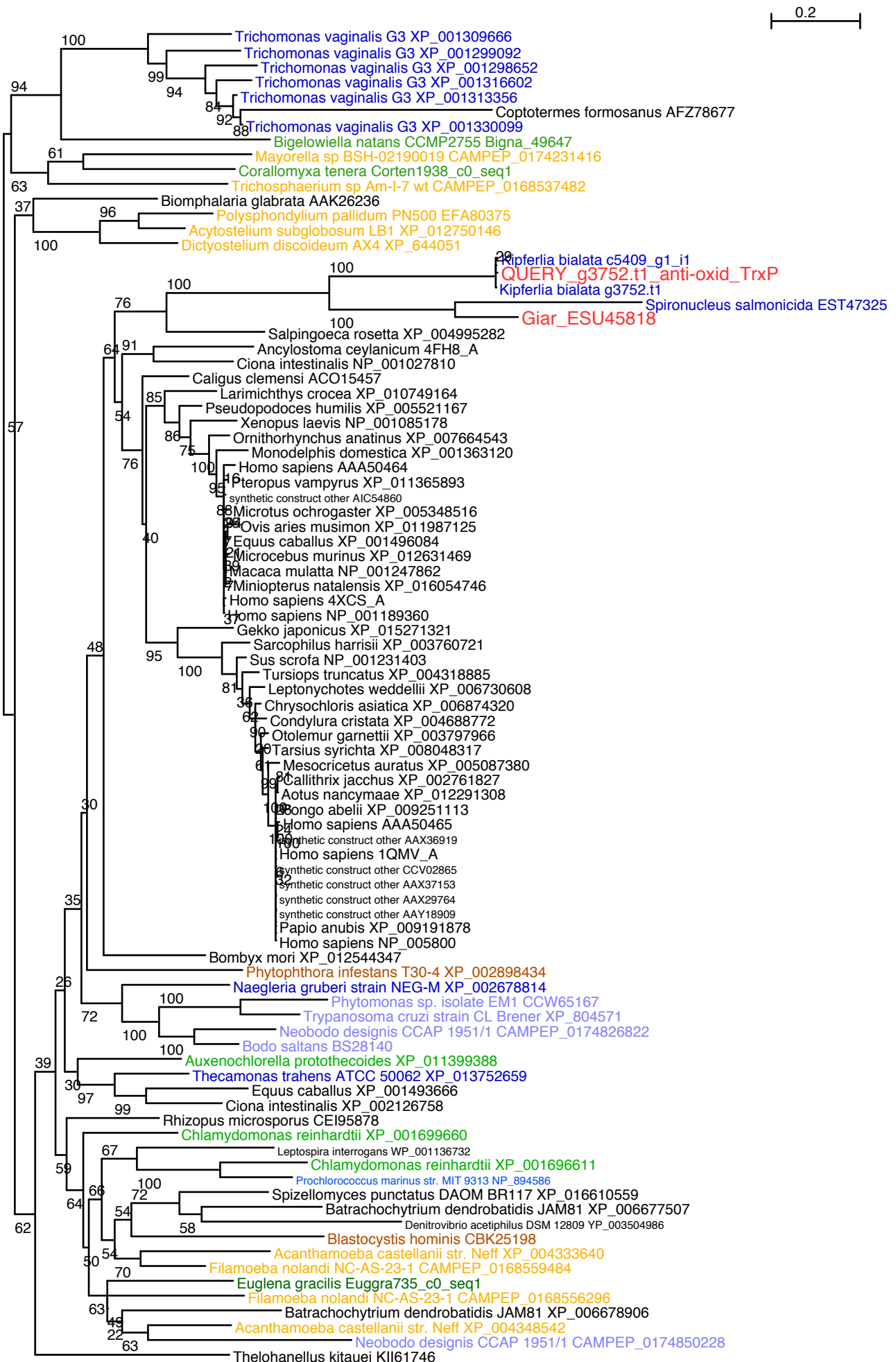

0.2

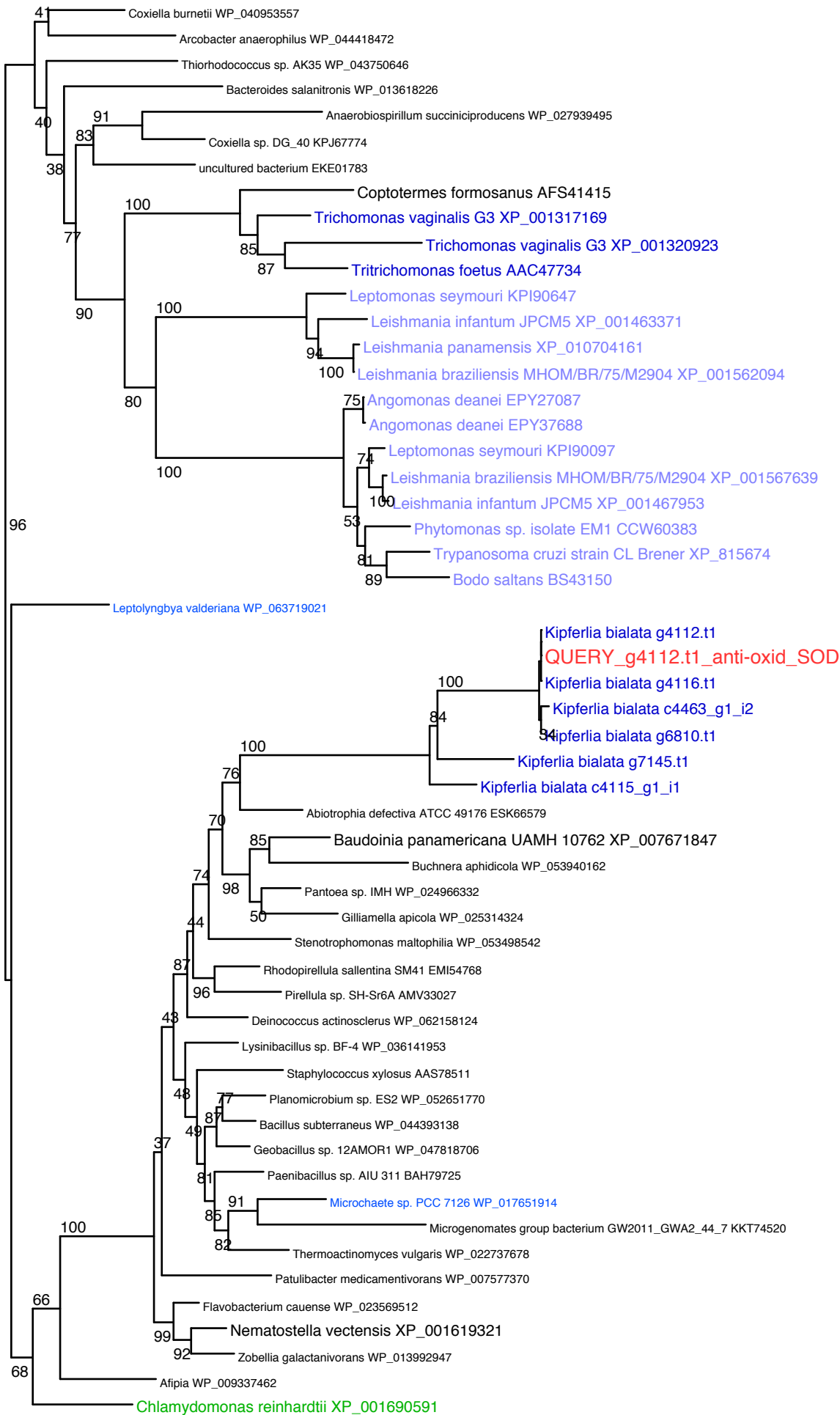

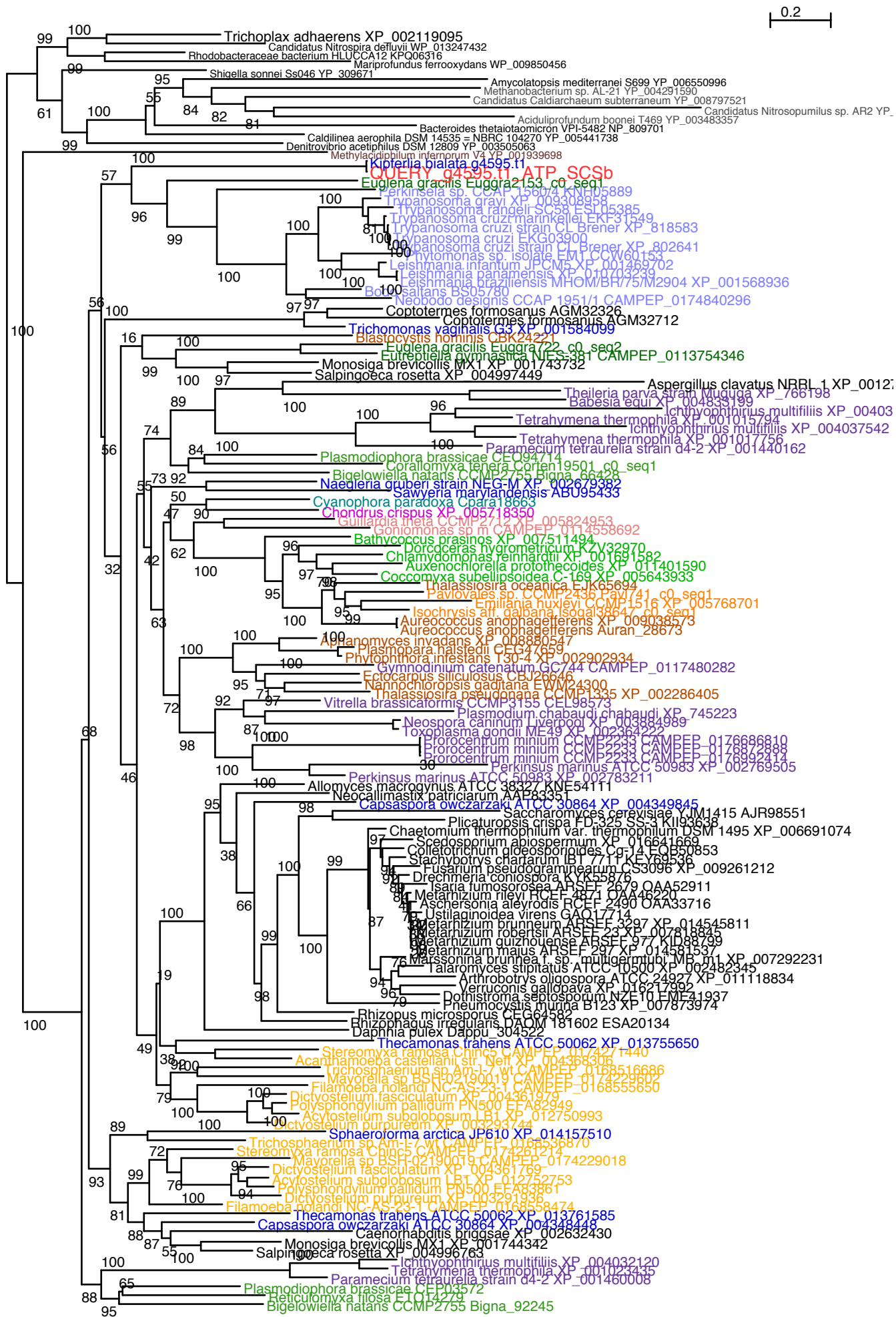

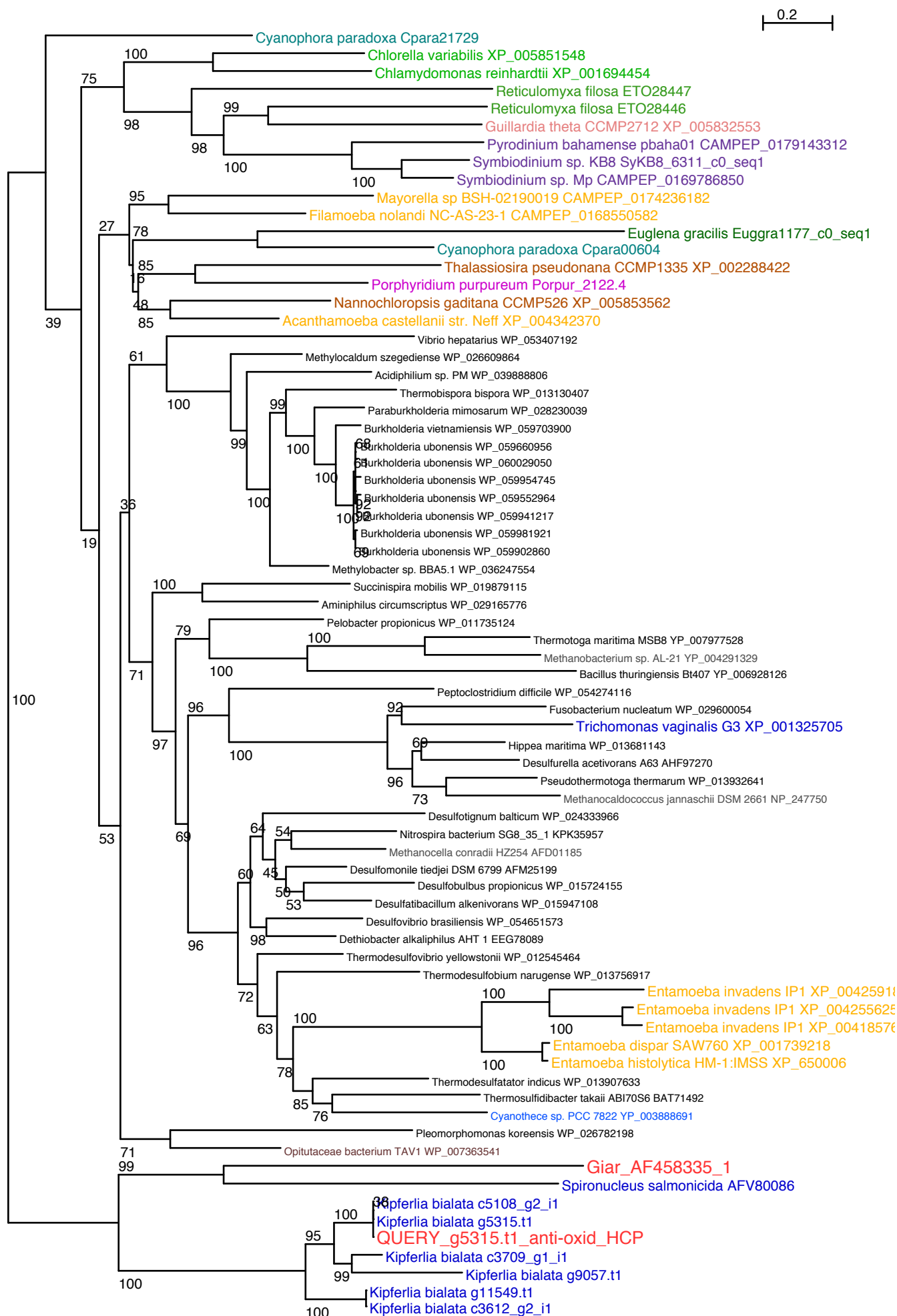

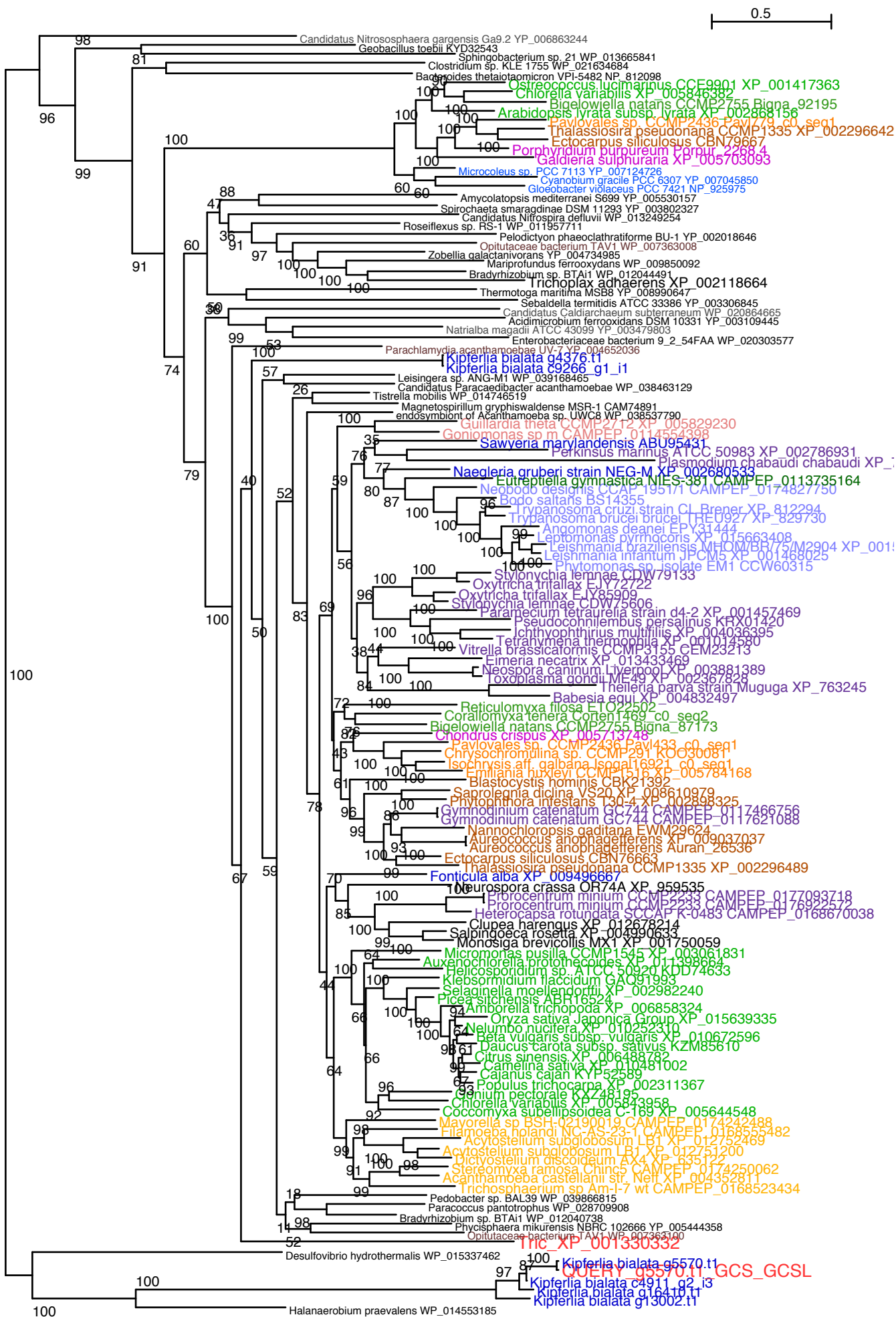

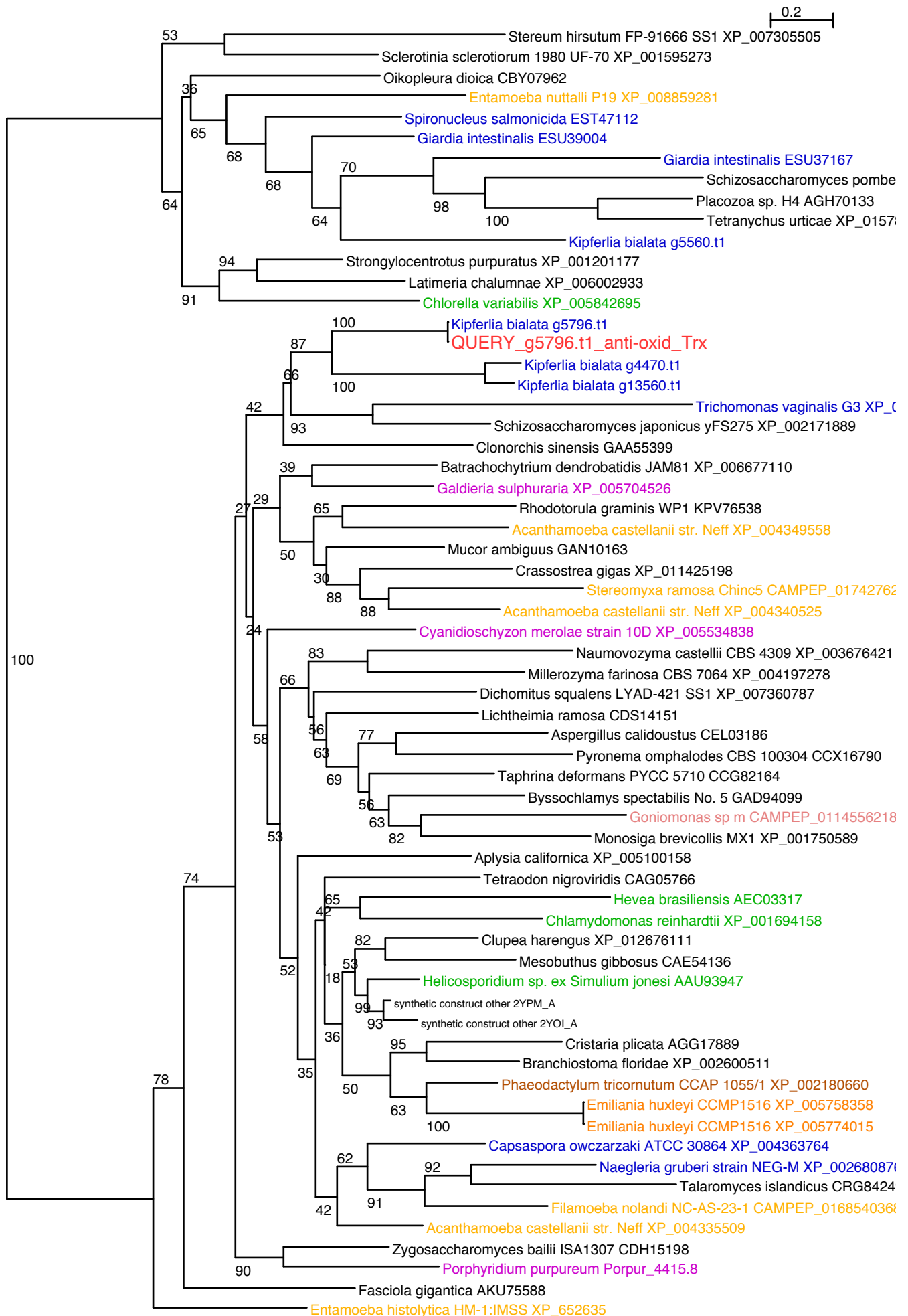

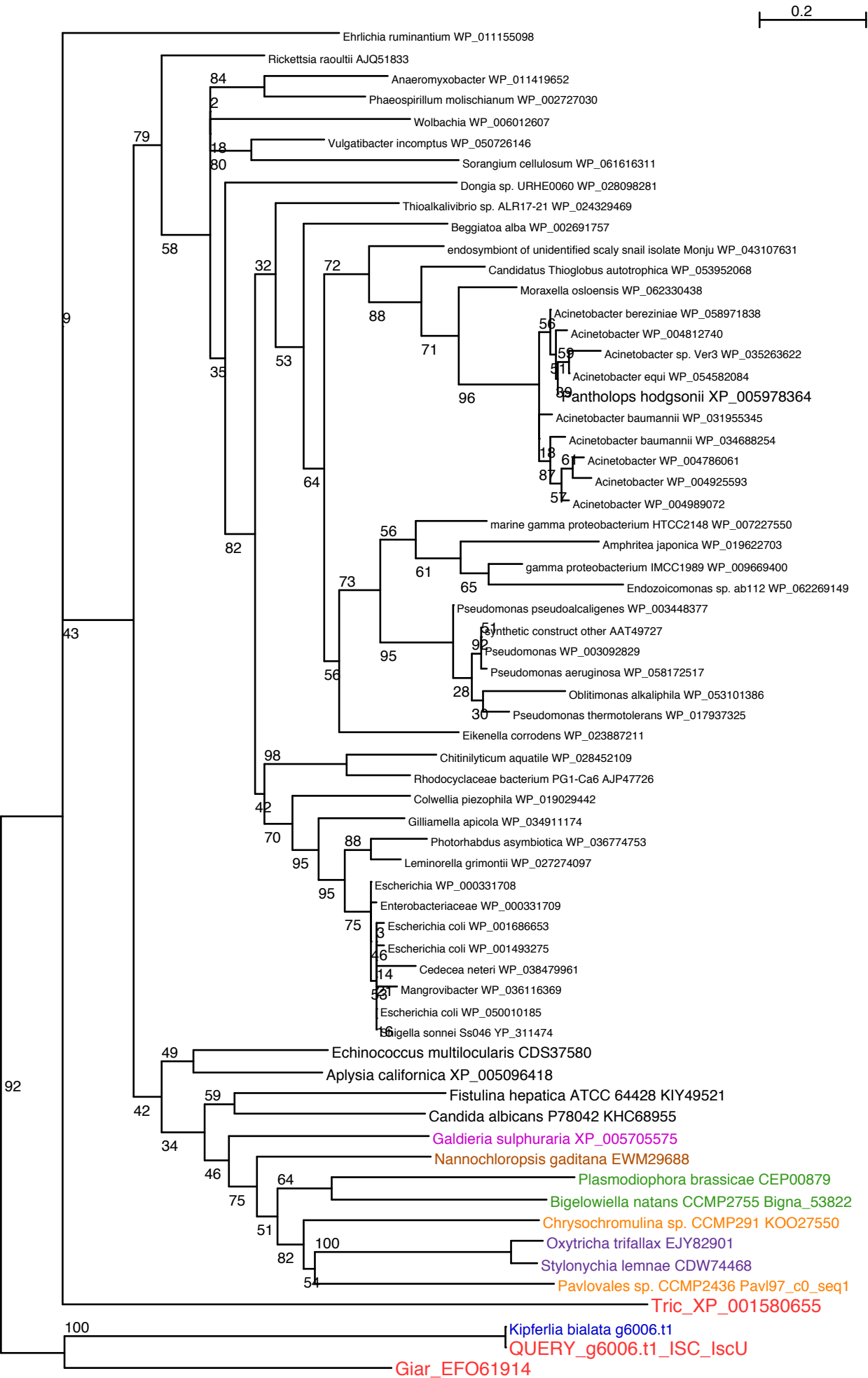

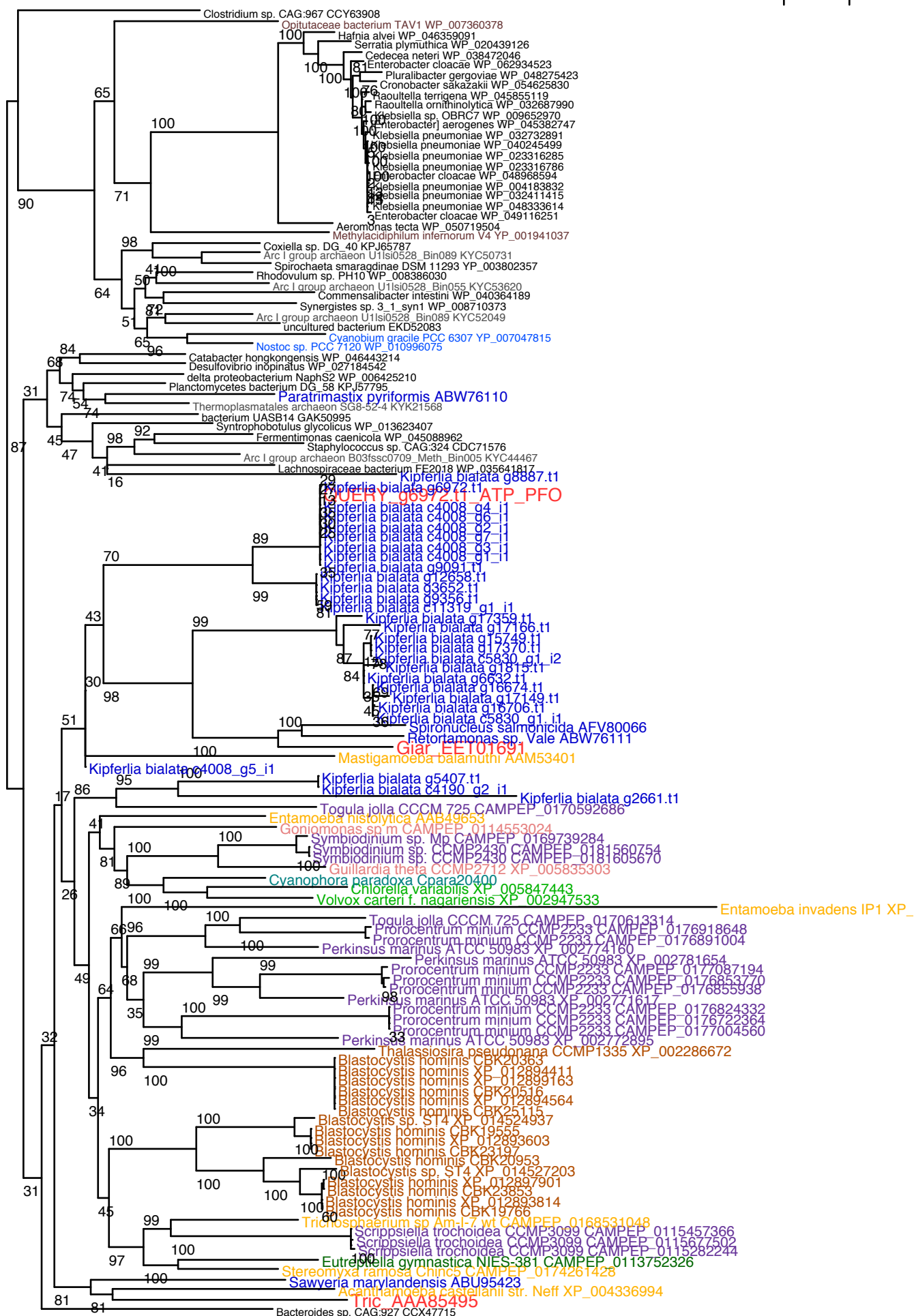

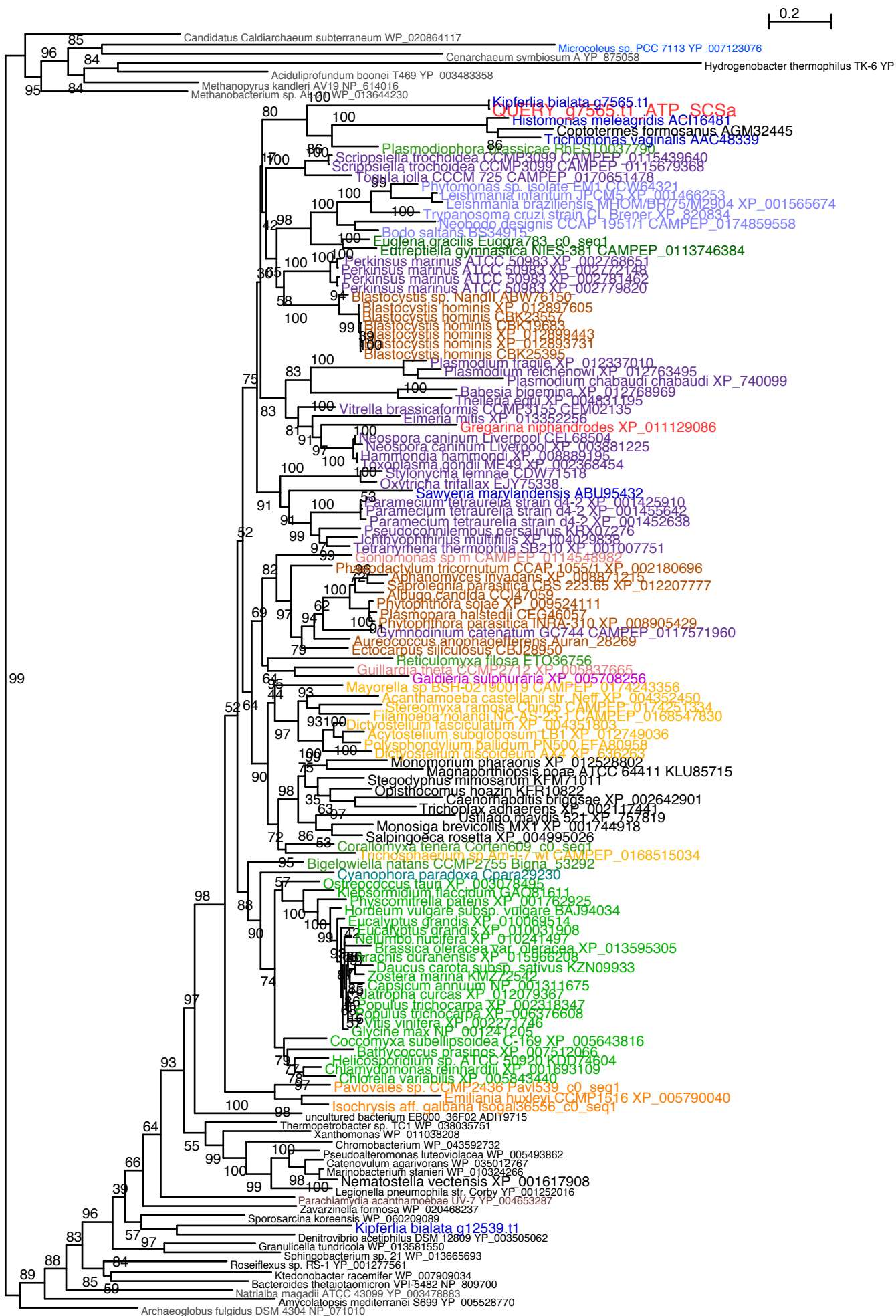

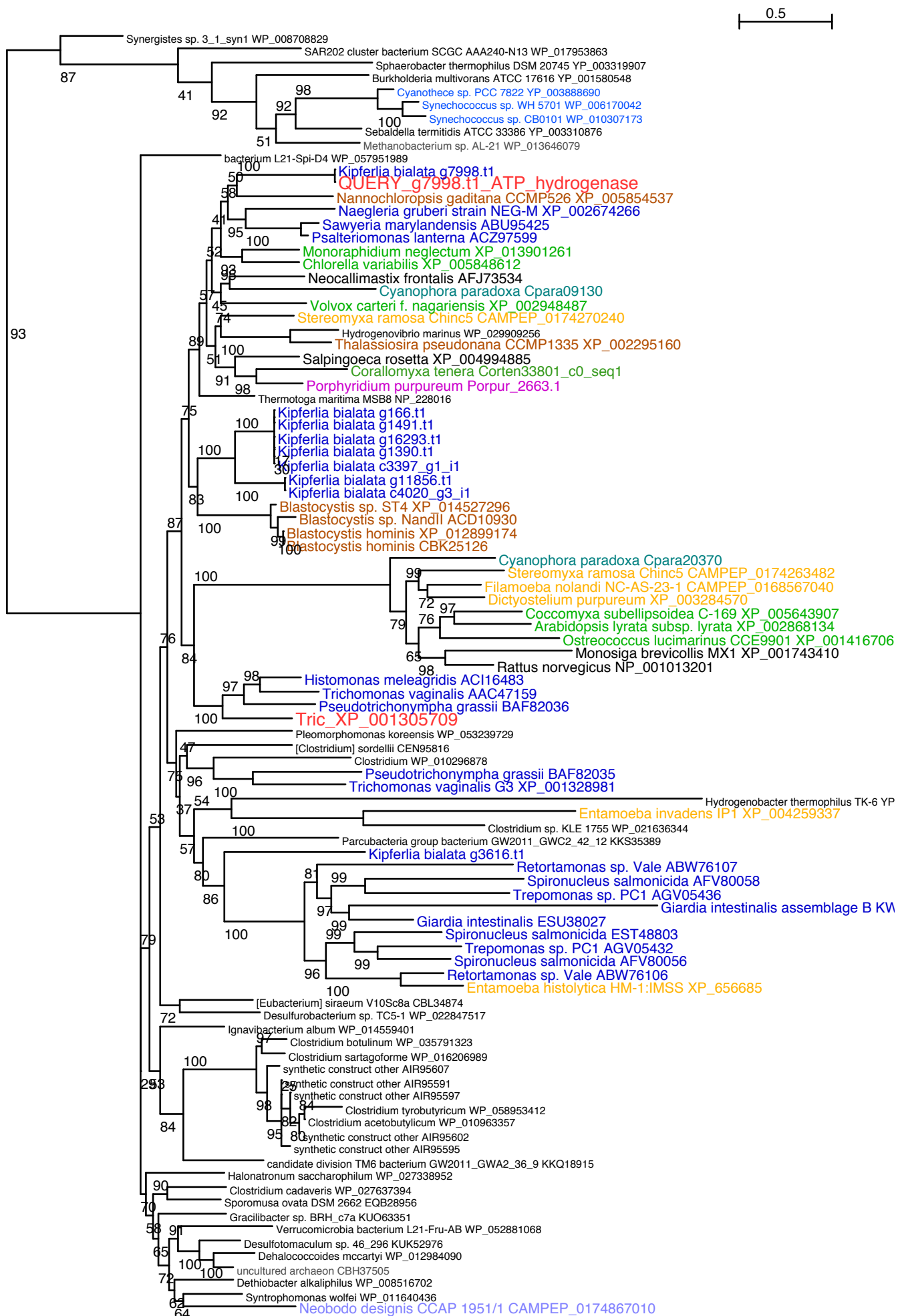

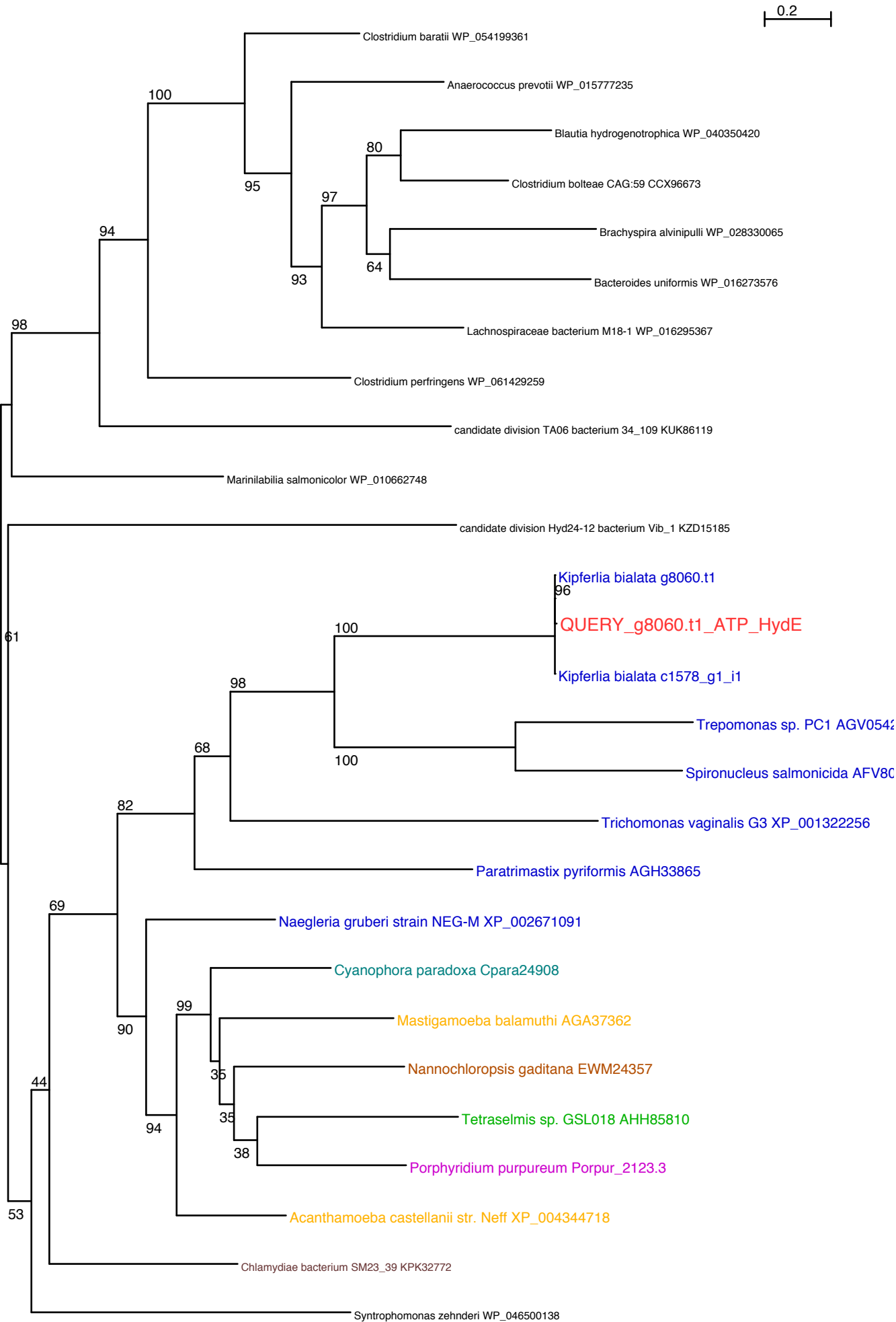

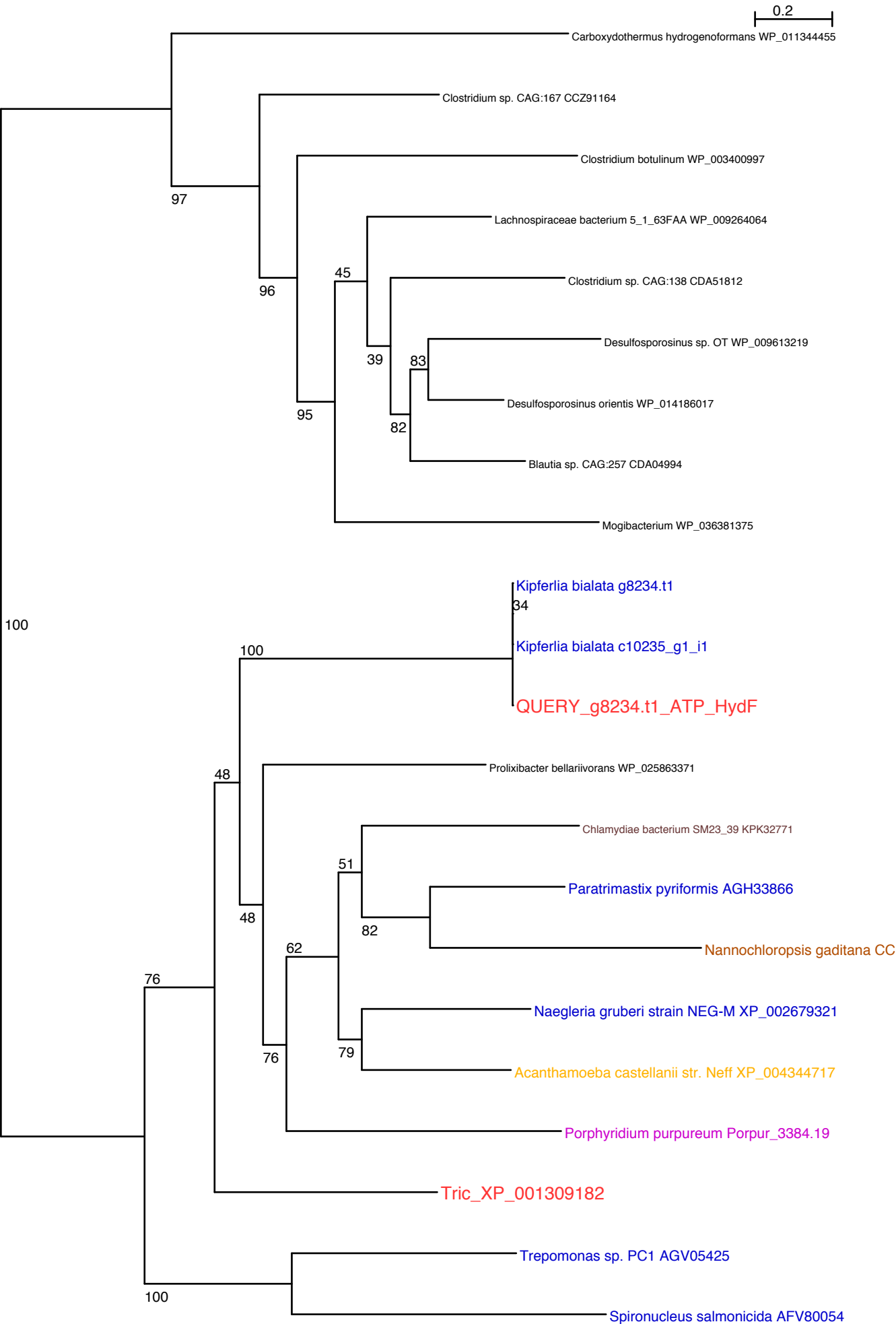

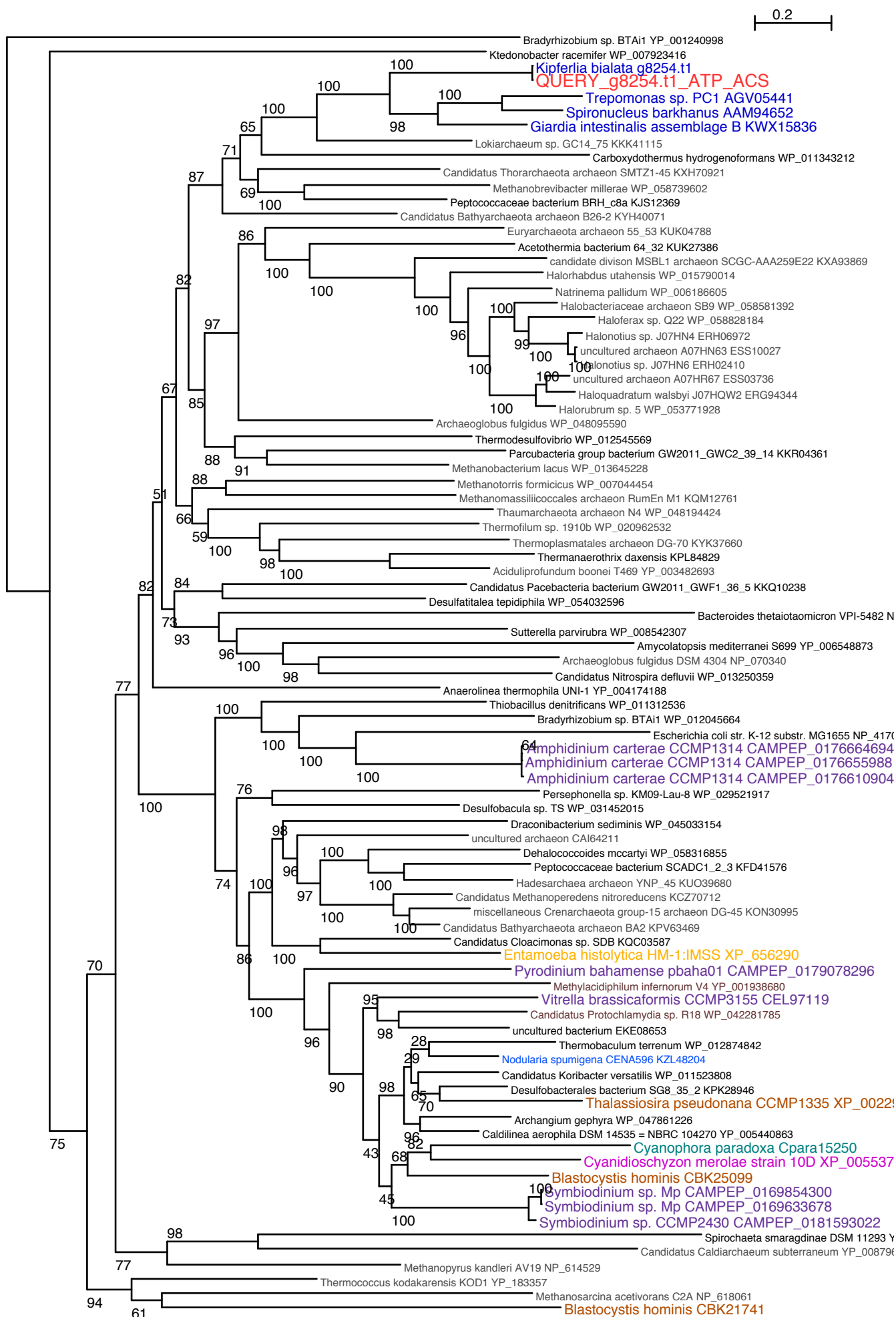

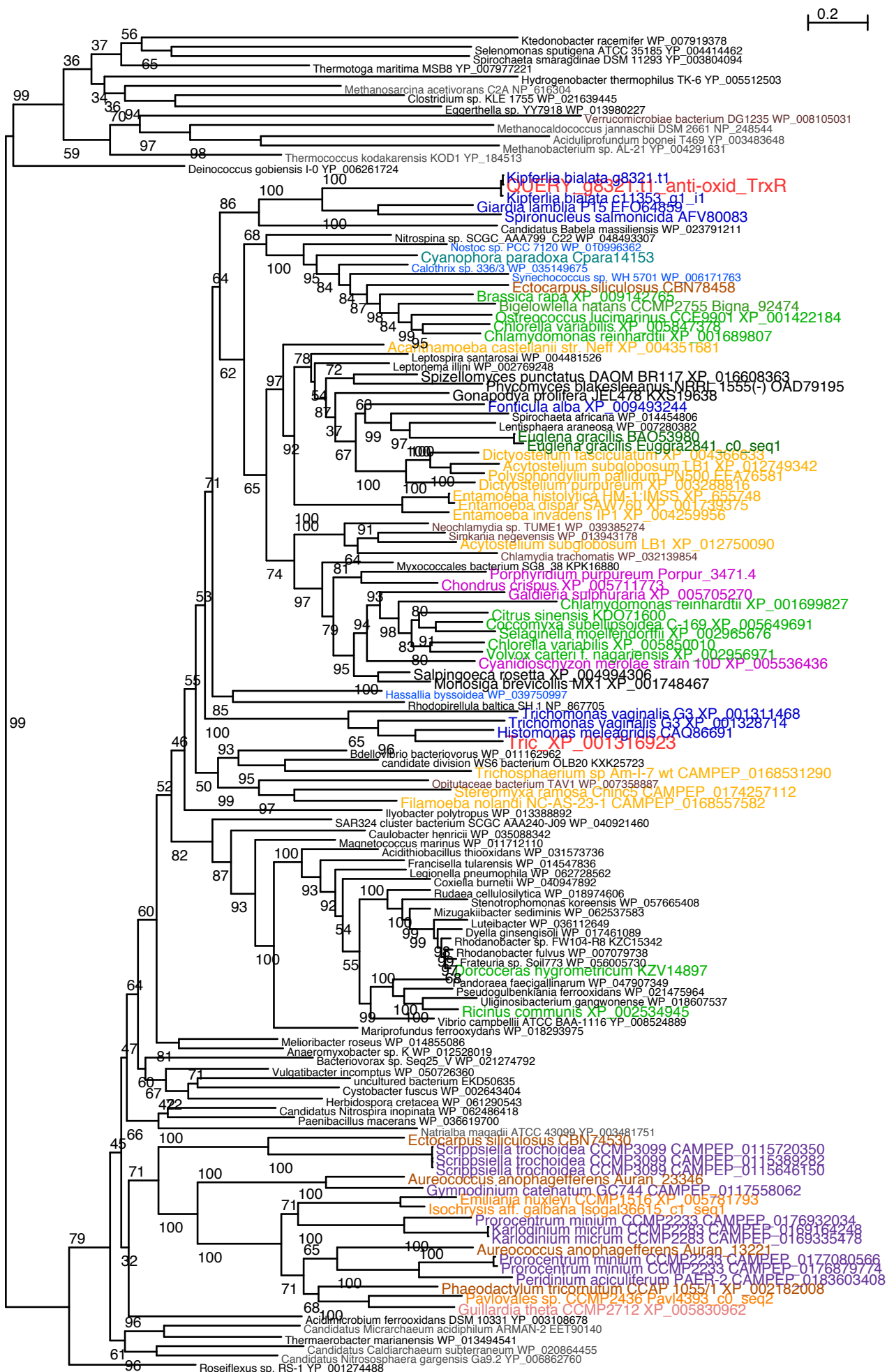

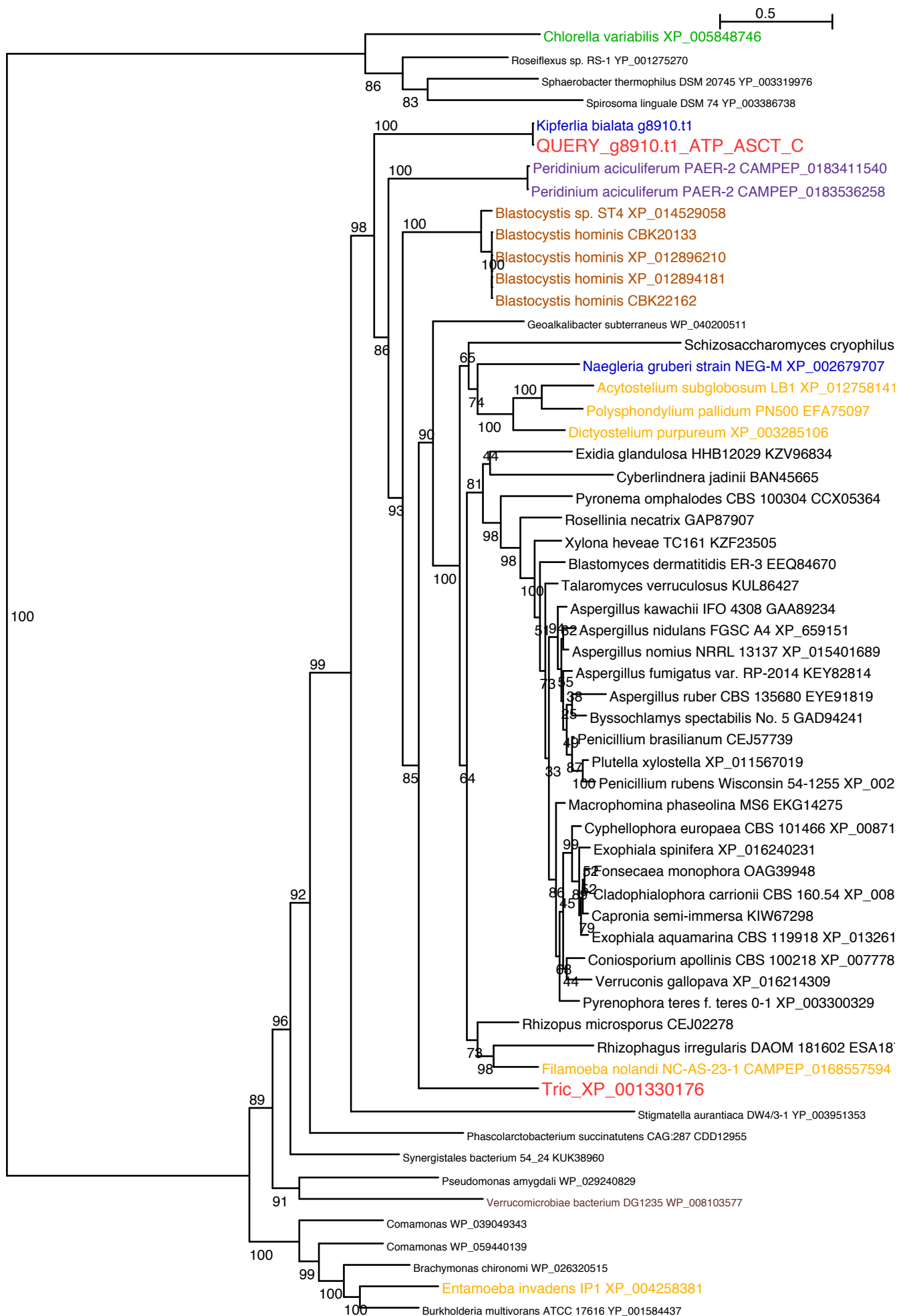

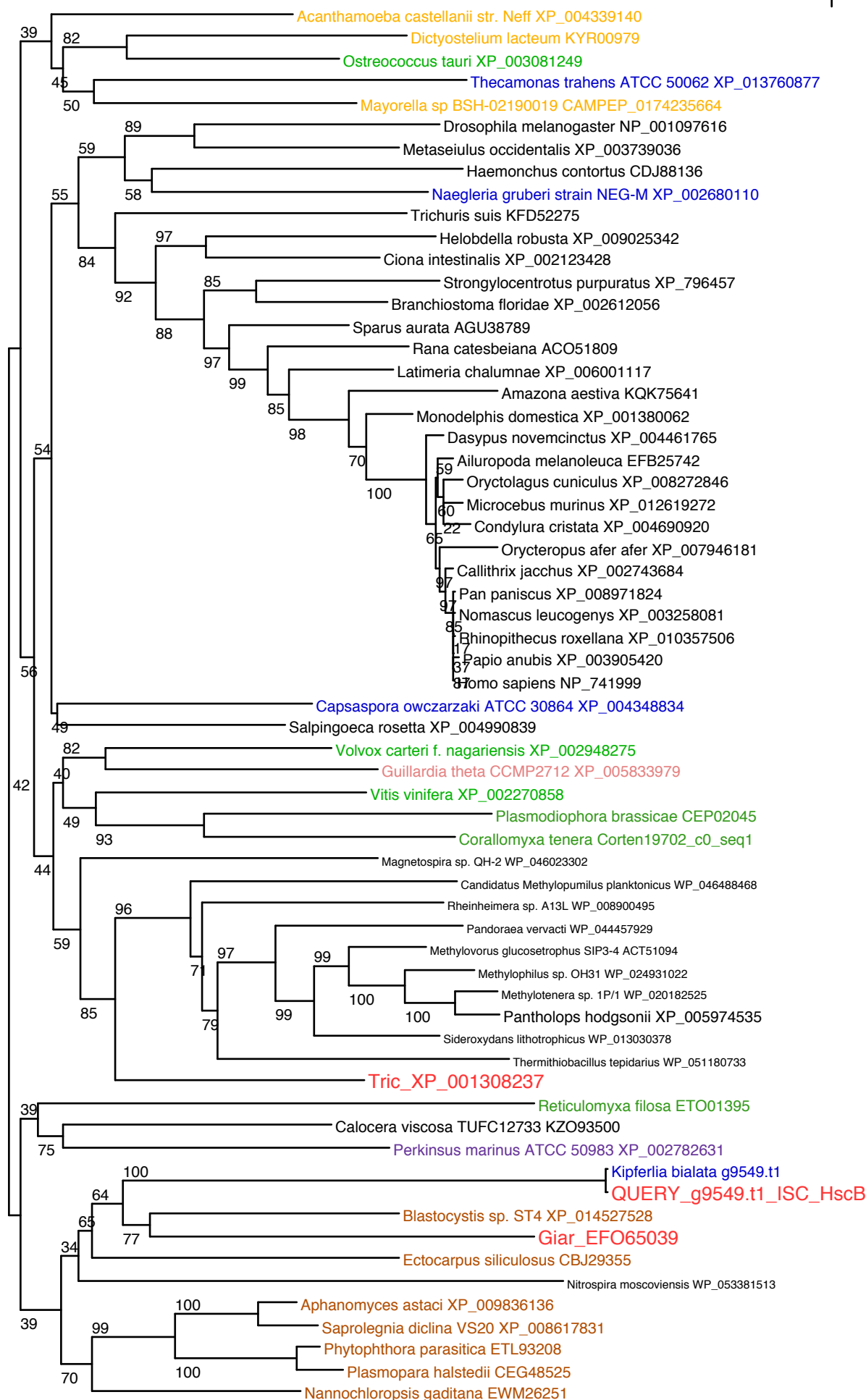

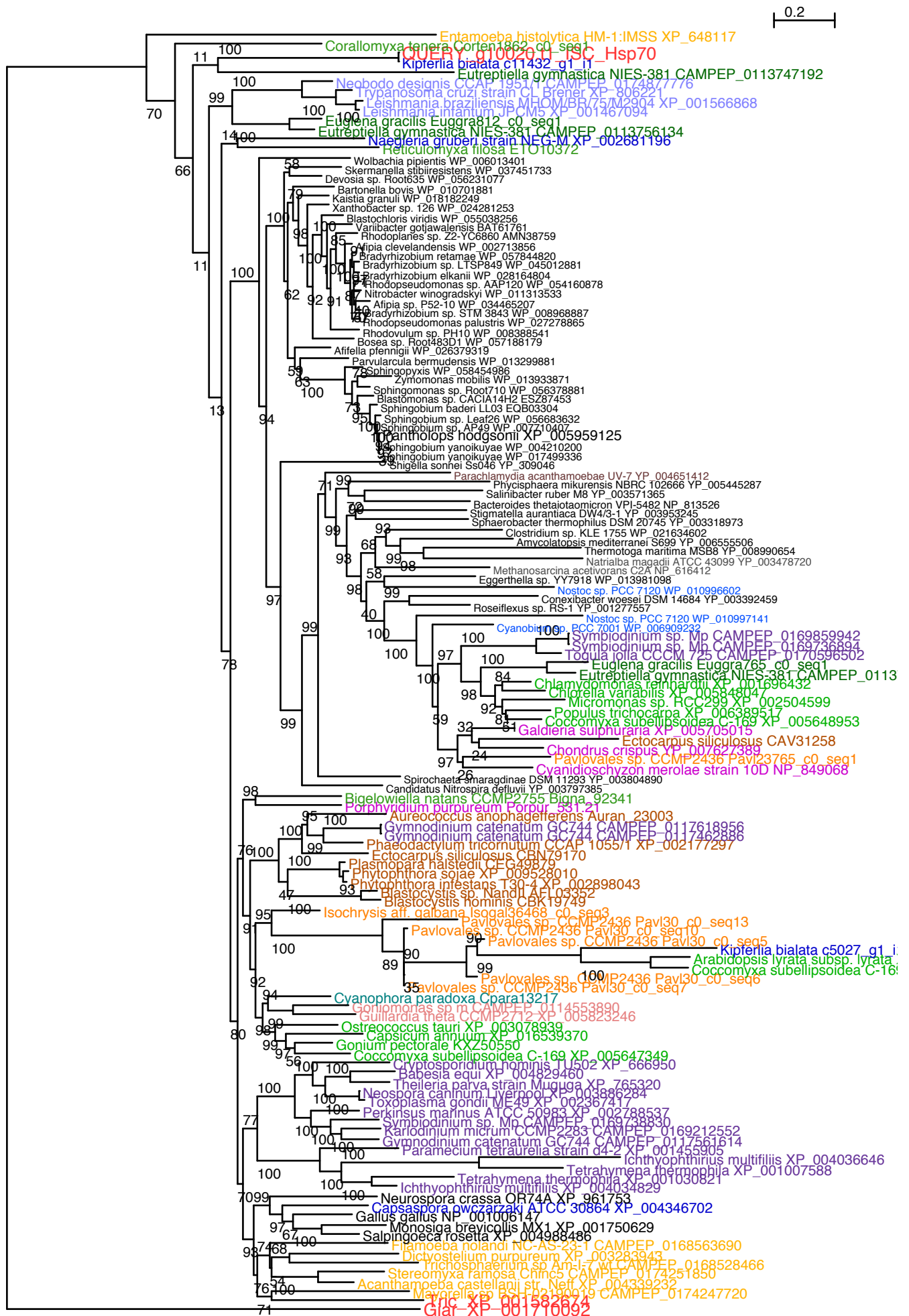

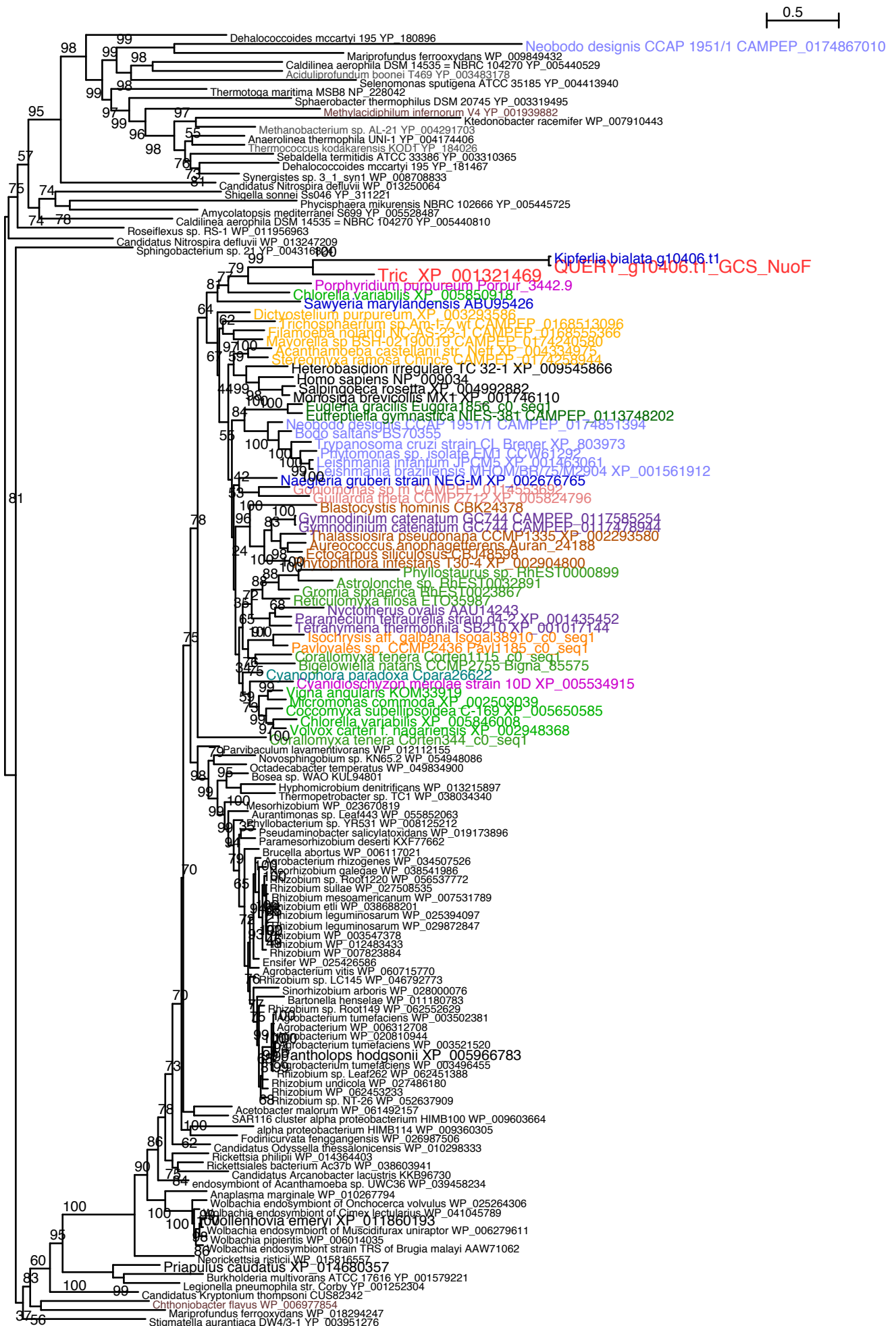

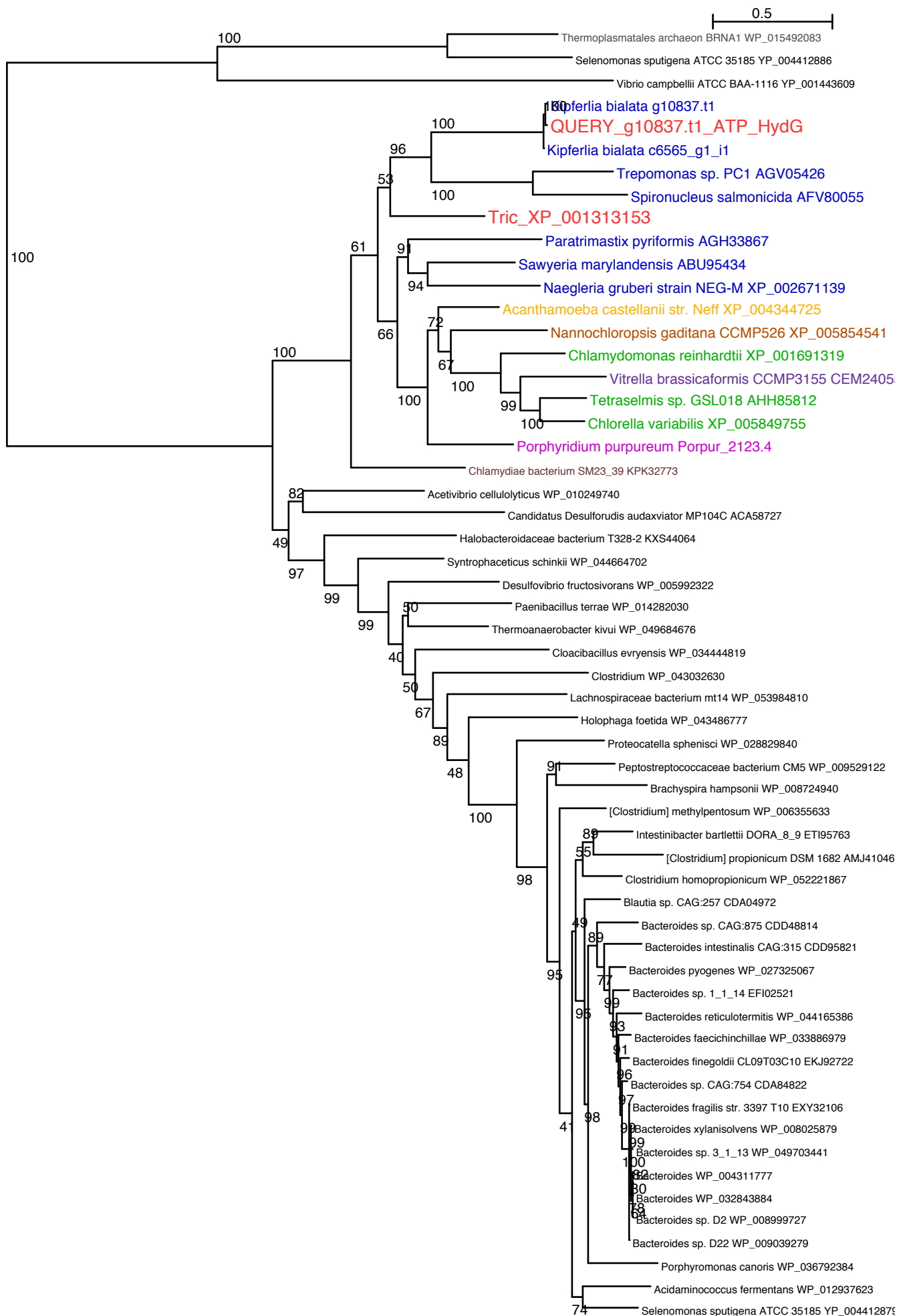

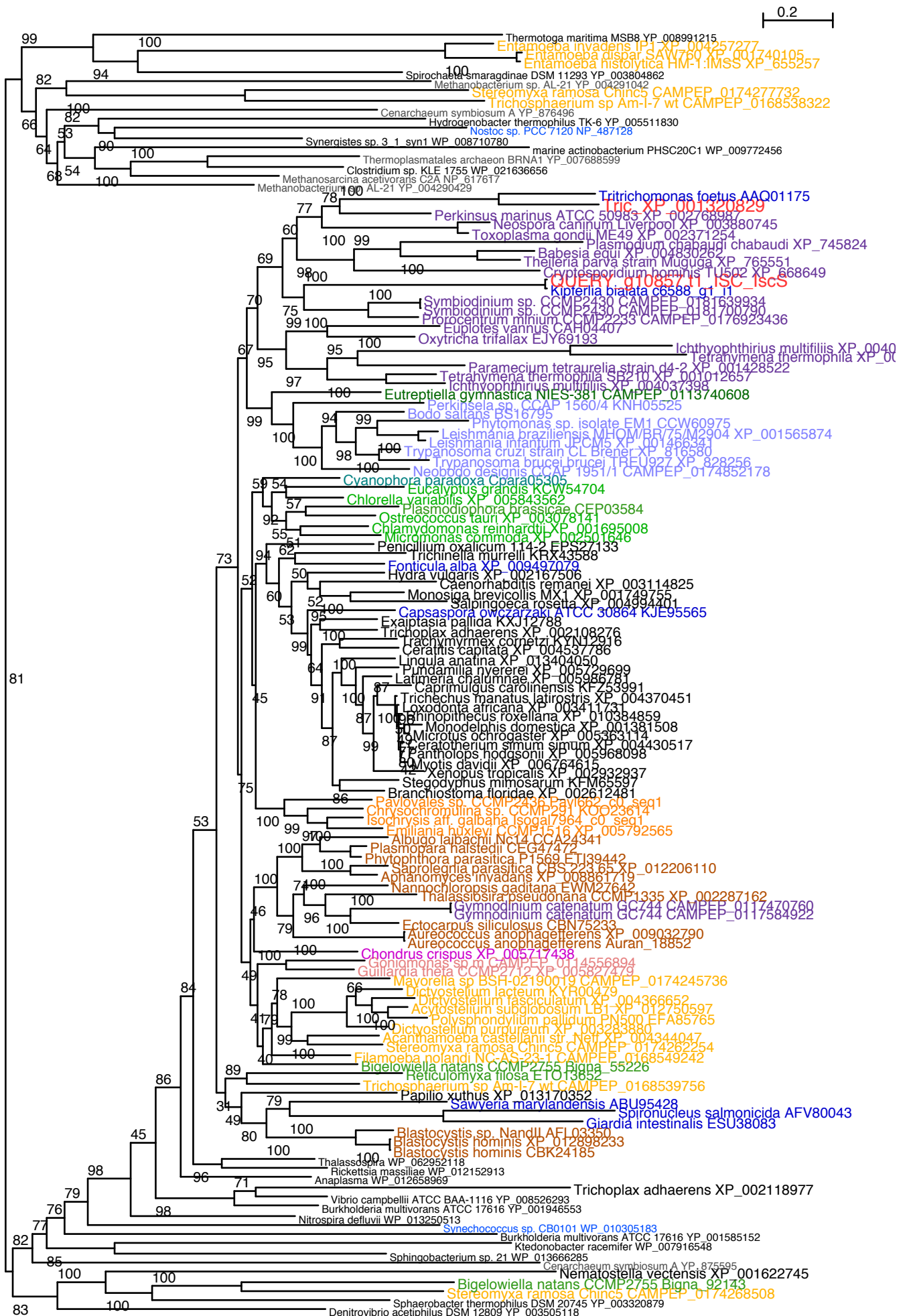

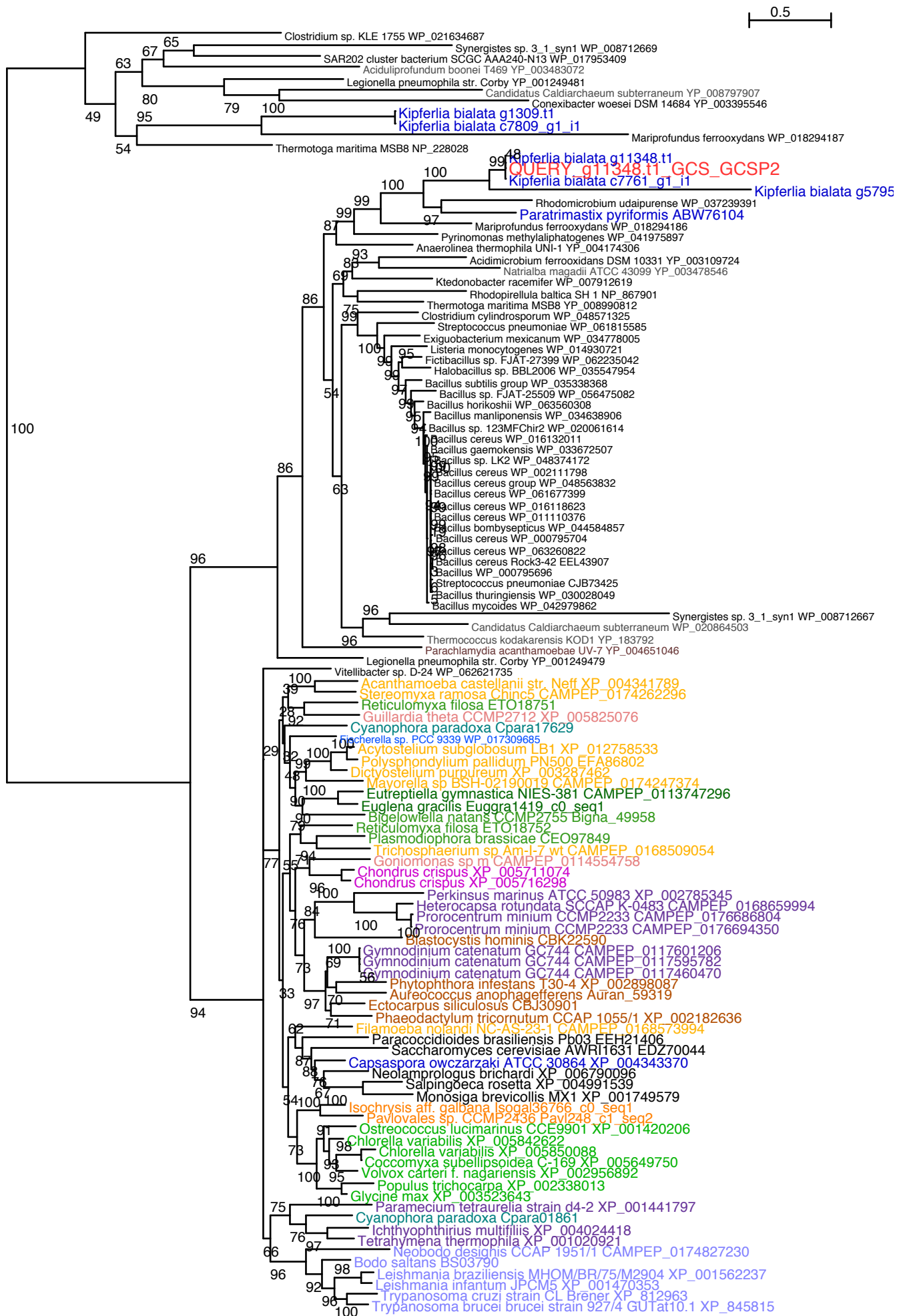

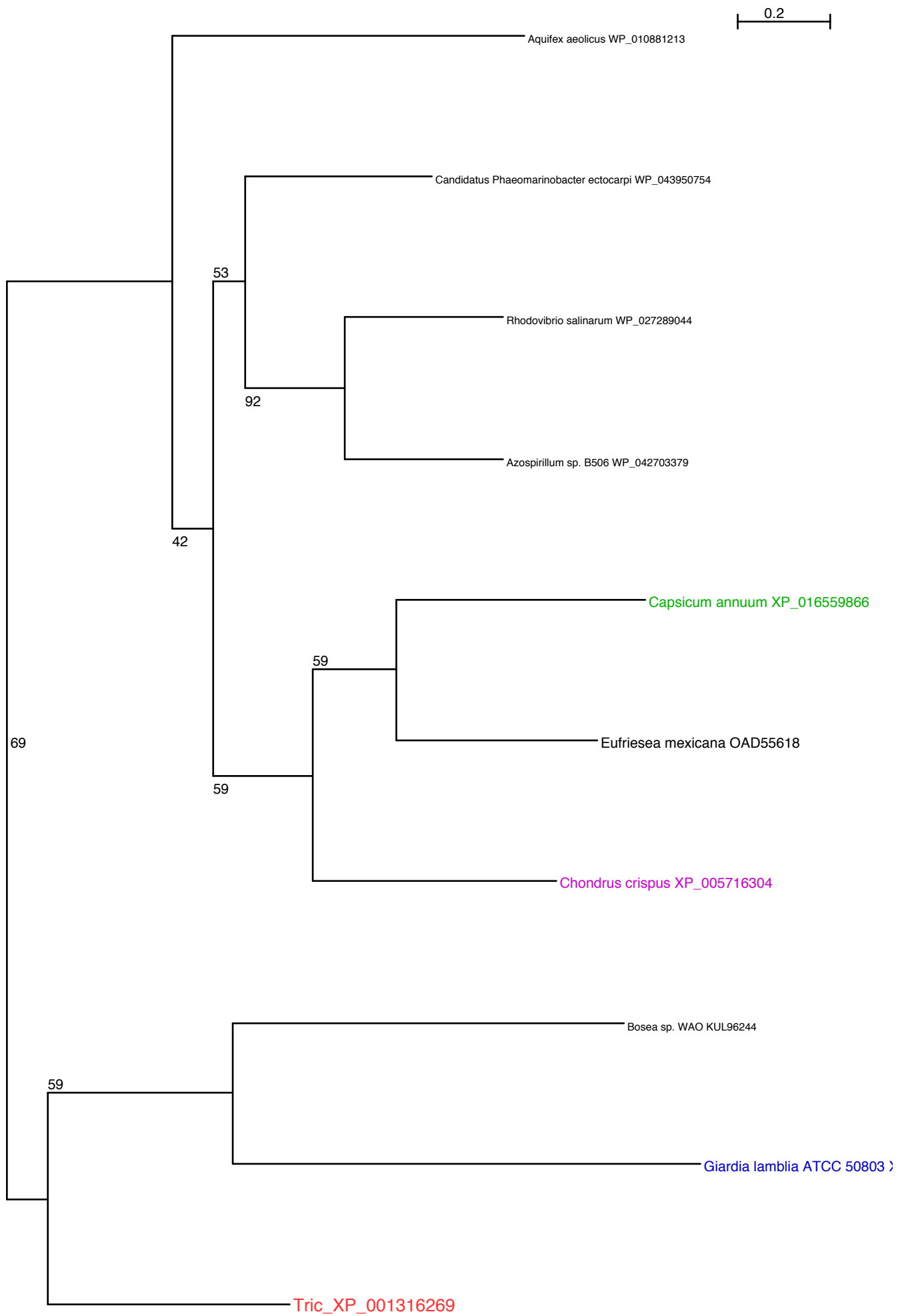

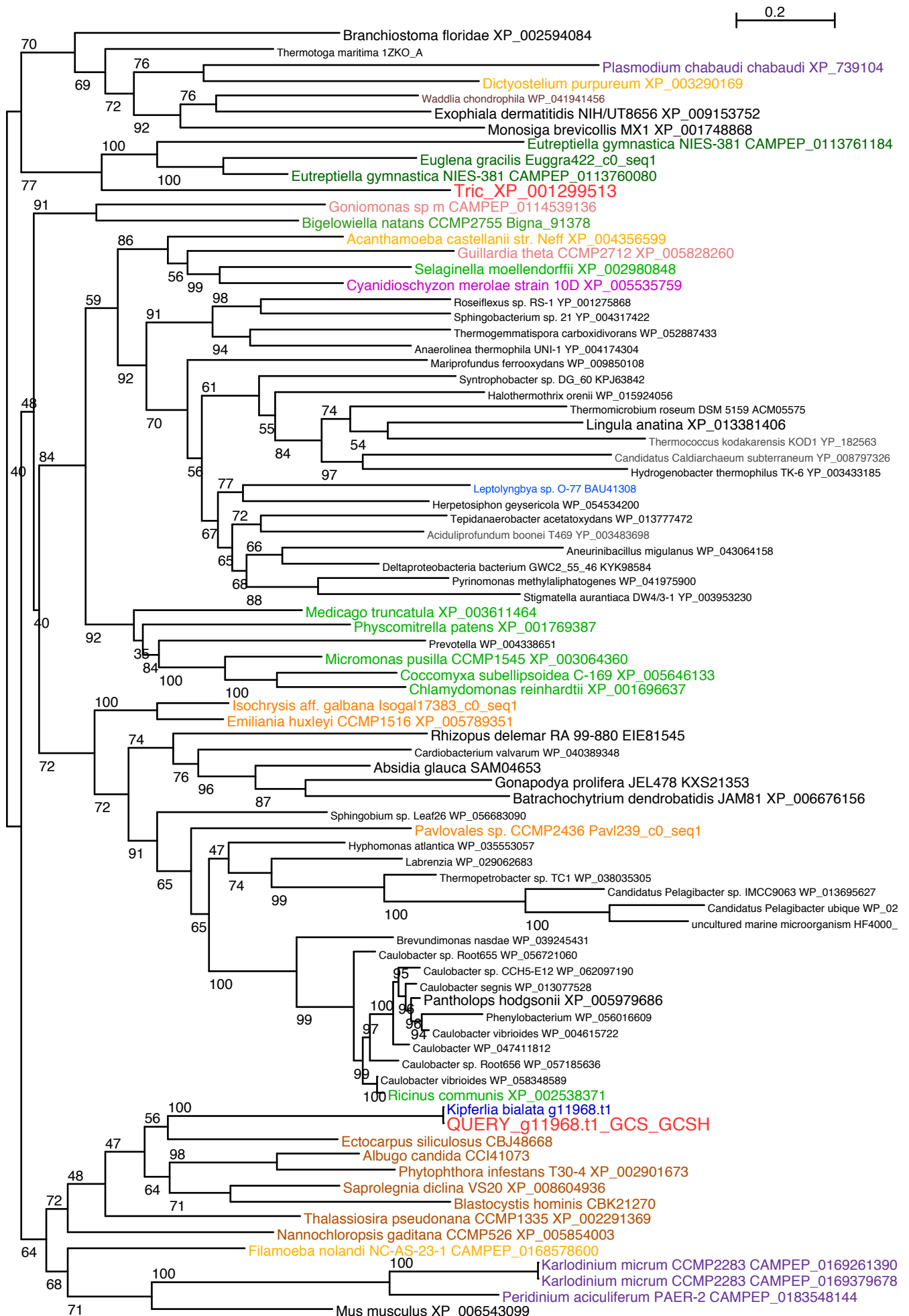

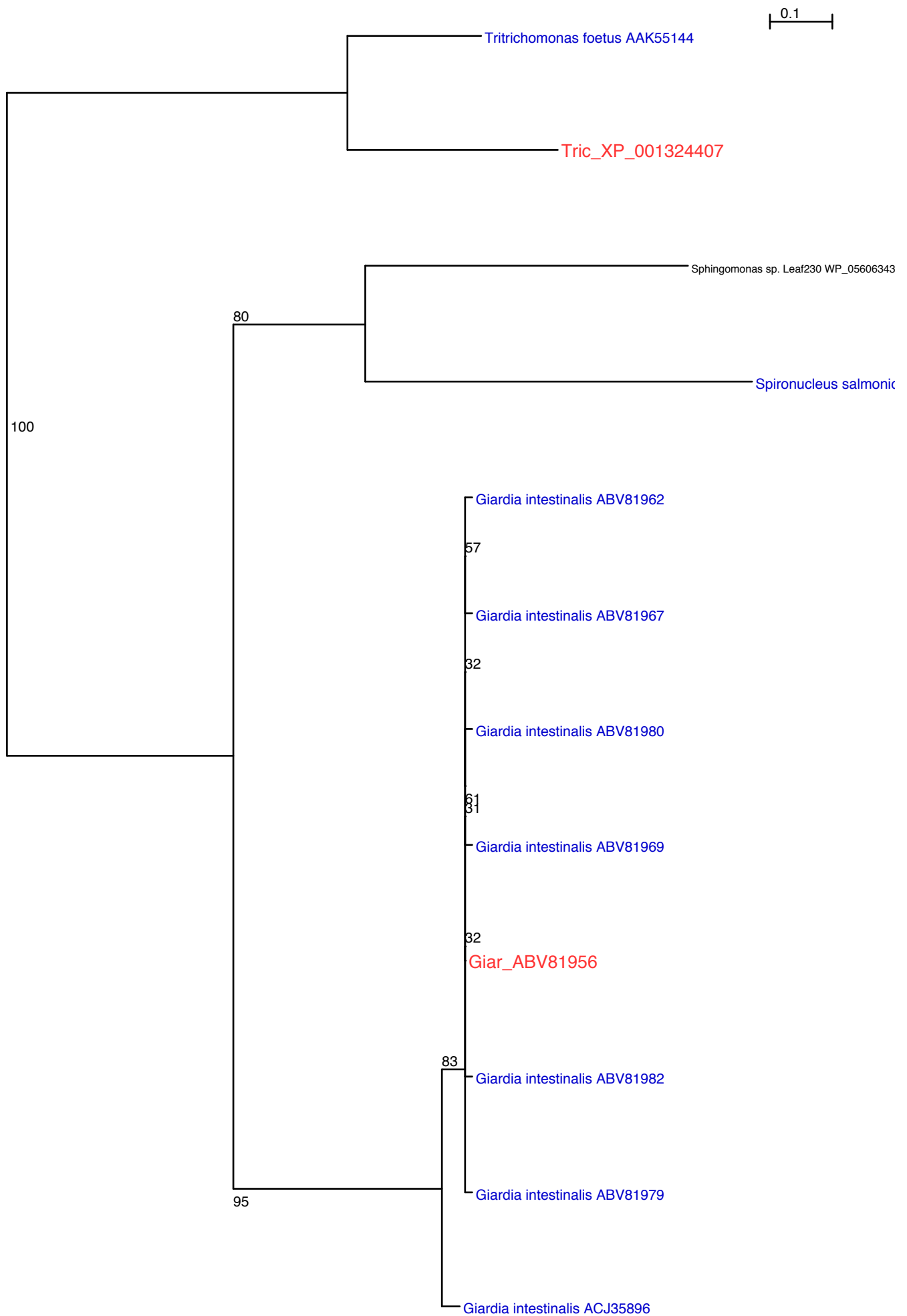

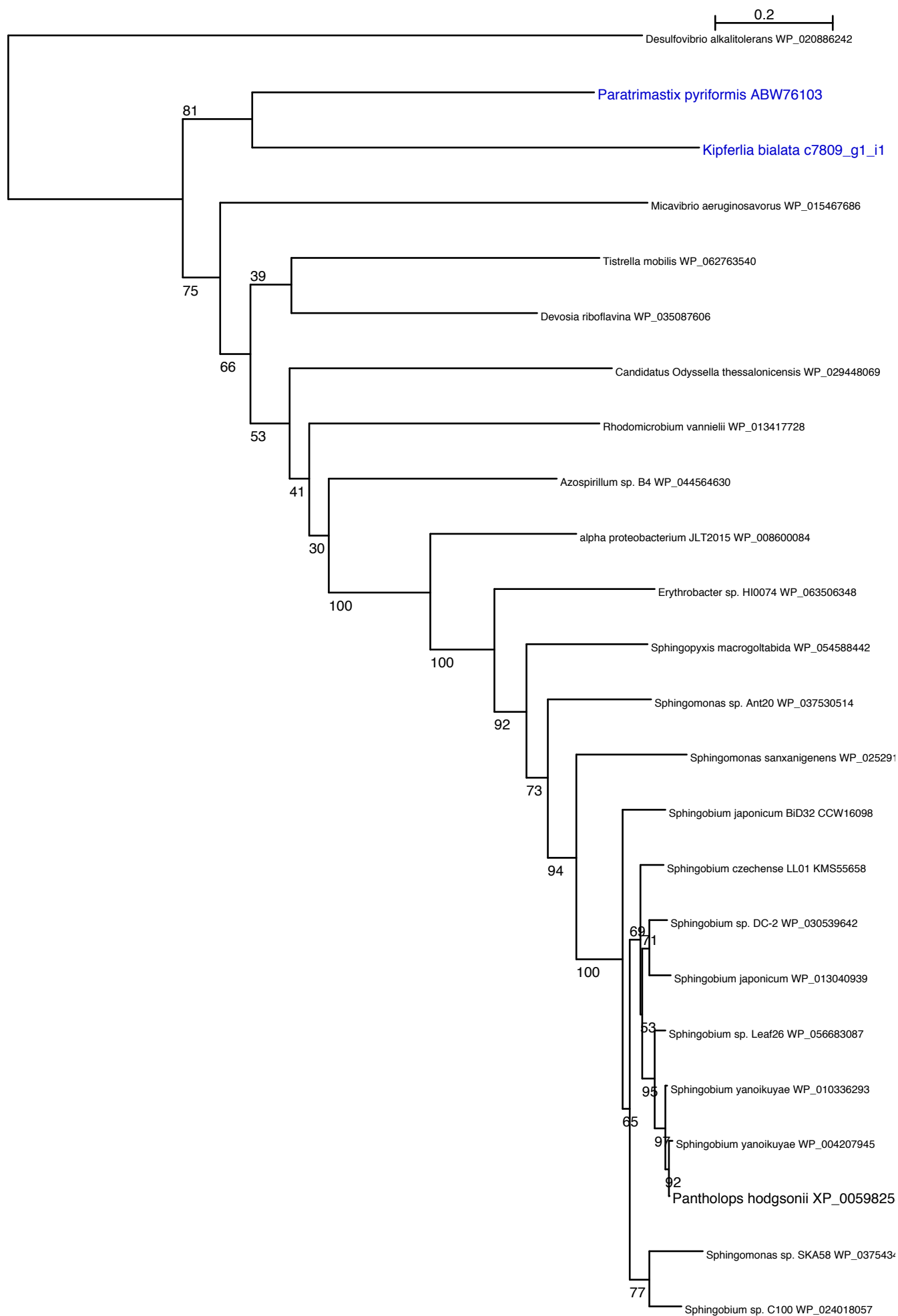

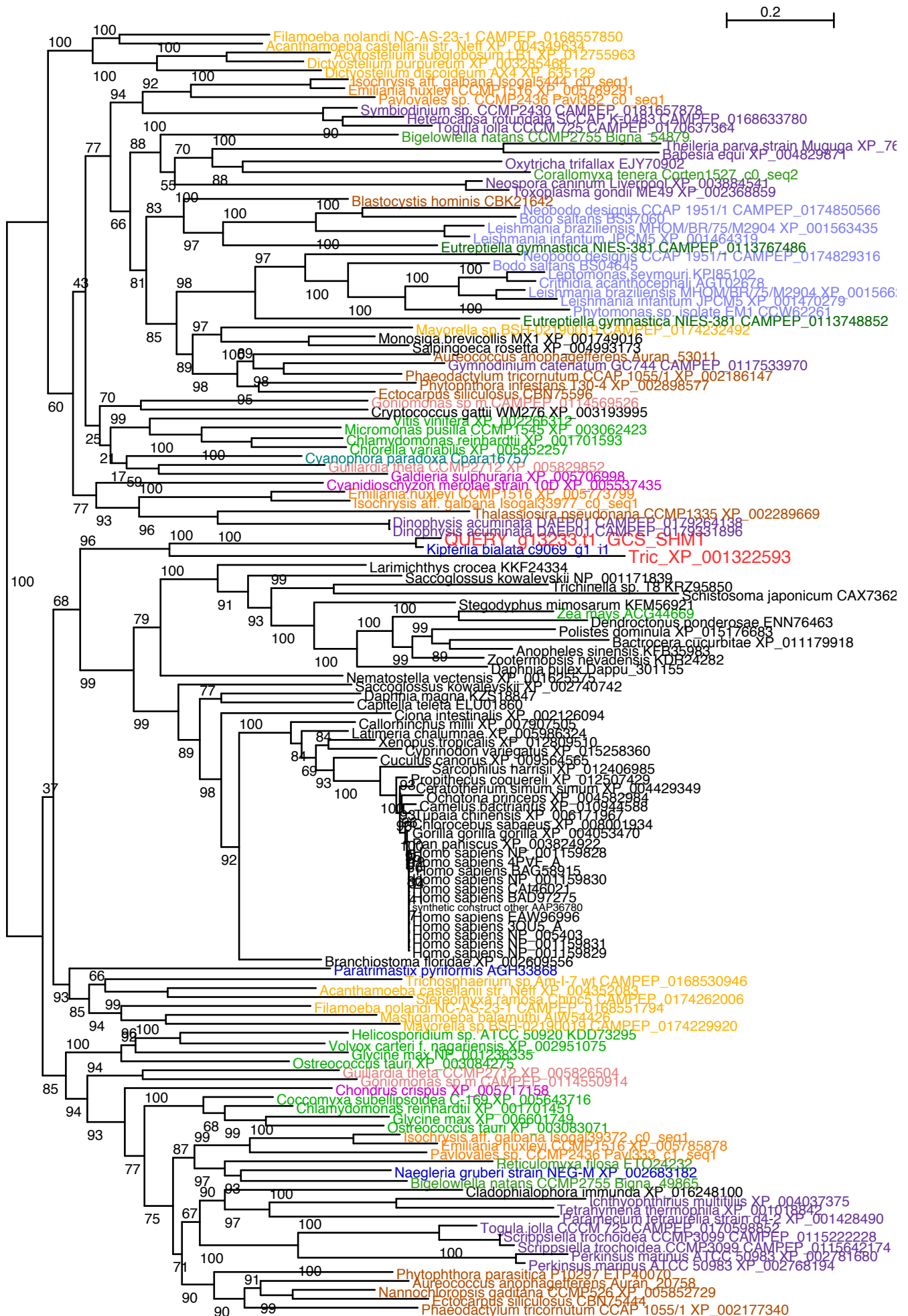

Supplement: S2 Fig — (PDF) [file pone.0194487.s002.pdf]
