# Supplementary material for: The draft genome of Kipferlia bialata reveals reductive genome evolution in fornicate parasites
Source: PLoS One. 2018 Mar 28;13(3):e0194487. doi: 10.1371/journal.pone.0194487 (PMC5874029; doi:10.1371/journal.pone.0194487)
Supplement: S1 Table — The left column indicates the protein IDs of K. bialata ATP transporter candidates. The second left column shows the number of transmembrane domains predicted by TMHMM 2.0c. Remaining columns display results of the tblanstn search. Each of the query proteins from human or mouse used for the search is shown in the top cell. log 10 of the e-value for the hit between the query and each of the K. bialata proteins is shown in the corresponding cell. Only the hits with e-value < 1e-15 are shown. (PDF) [file pone.0194487.s003.pdf]

S1 Table. Predictable ATP transporters in *Kipferlia bialata*. The left column indicates the protein IDs of *K. bialata* ATP transporter candidates. The second left column shows the number of transmembrane domains predicted by TMHMM 2.0c. Remaining columns display results of the tblanstn search.Each of the query proteins from human or mouse used for the search is shown in the top cell. log 10 of the e-value for the hit between the query and each of the *K. bialata* proteins is shown in the corresponding cell. Only the hits with e-value < 1e-15 are shown.

	#TM domain	ABCB10_Homo	ABCB10_Mus	ABCB7_HUMAN	ABCB7_MOUSE	ABCB8_HUMAN	ABCB8_MOUSE	ABCB6_HUMAN	ABCB6_MOUSE
c1365_g2_i1	2	51	47	43	41	47	47	48	49
c4639_g3_i1	5	34	30	29	29	26	28	23	26
c5517_g20_i2	5	31	29	29	29	30	30	29	-
c5517_g20_i1	5	30	28	29	29	30	30	29	-
c5517_g21_i1	9	18	29	18	17	17	18	32	17
c5142_g1_i1	9	18	18	21	20	16	17	15	17
c5142_g1_i4	9	18	18	21	20	16	17	15	17
c5142_g1_i3	9	18	18	21	20	16	17	15	17
c5751_g28_i1	0	15	15	18	16	-	-	-	-
c5751_g28_i2	0	-	-	17	-	-	-	15	17
c5751_g22_i1	9	-	-	15	-	-	-	16	16
c339_g1_i1	6	16	-	-	-	16	-	-	-
c4882_g1_i1	7	-	-	-	-	-	18	-	-
c4882_g1_i2	13	-	-	-	-	-	18	-	-
c5708_g22_i1	0	18	-	-	-	-	-	-	-
c6255_g1_i1	0	-	-	-	-	-	-	-	16
c5032_g1_i3	6	-	-	-	-	-	-	15	-
c5032_g1_i1	8	-	-	-	-	-	-	15	-
c5334_g1_i2	6	-	-	-	-	-	-	-	15
c5870_g1_i8	5	15	-	-	-	-	-	-	-
